# Supplementary material for: Coordinative Changes in Metabolites in Grape Cells Exposed to Endophytic Fungi and Their Extracts
Source: Molecules. 2022 Aug 29;27(17):5566. doi: 10.3390/molecules27175566 (PMC9458220; doi:10.3390/molecules27175566)
Supplement: Supplementary file 1 [file molecules-27-05566-s001.zip › molecules-1860993-supplementary.pdf]

Table S1. Raw data of the metabolites profiled in all grape cell samples of all treatments.

(The mix samples for quality control)

Index	Compounds	Class I	mix01	mix02	mix03	mix04
mws0470	Methylmalonic acid*	Organic acids	34682000	36120000	37947000	35493000
mws0192	Succinic acid*	Organic acids	33338000	33487000	35523000	32819000
pme0230	Adenosine	Nucleotides and derivatives	22259000	21206000	22794000	22534000
pme0021	L-Phenylalanine	Amino acids and derivatives	19193000	19142000	18993000	19134000
mws0277	Kinic acid	Organic acids	17743000	18772000	18412000	18449000
pmb0789	Pyridoxine-5'-O-glucoside	Others	16686000	18223000	17274000	17767000
mws0216	Trans-4-Hydroxy-L-proline	Amino acids and derivatives	19023000	16160000	16667000	18581000
mws0281	Citric Acid	Organic acids	18170000	16161000	14860000	15636000
pmc0274	6-Methylmercaptopurine	Nucleotides and derivatives	14149000	13987000	13997000	14518000
Rfmb324	Trigonelline	Alkaloids	10740000	11415000	10034000	11008000
pme0006	L-Proline	Amino acids and derivatives	9767100	11378000	9693400	9311600
pmn001578	Hexadecanoic acid	Lipids	9458400	8024100	7853800	7093800
mws0119	Myristic Acid	Lipids	7206100	7541300	7155200	6780600
pmp001287	N-Benzylmethylene isomethylamine	Alkaloids	6255700	6882400	6006800	6471400
pme1474	5'-Deoxy-5'-(methylthio)adenosine	Nucleotides and derivatives	6242200	6593600	7375500	7245100
mws1489	Stearic Acid	Lipids	5175100	5755800	6053900	5492300
mws0256	L-Valine*	Amino acids and derivatives	5922200	6376200	6067100	5753400
Zmhn001970	Piceid	Others	8143800	7945100	4427500	2824700
pmb0855	LysoPC 16:0	Lipids	5610000	5544700	5253900	5420700
mws0250	L-(-)-Tyrosine	Amino acids and derivatives	5250300	4934800	5041800	5124600
pme1383	Pyridoxine	Others	5095400	4916200	5424600	5144400

mws0366	γ -Linolenic Acid	Lipids	5358400	5417100	5232300	4772200
pmp001198	6-Deoxyfagomine	Alkaloids	4245100	4679600	4529700	4457300
pmb3101	2-Isopropylmalate	Organic acids	4548600	4544600	4277500	4217900
Hmsn000210	Methyl 7,10-hexadecadienoate	Lipids	3453400	3475300	3738400	3324400
pme2380	A-Ketoglutaric acid	Organic acids	3925600	3640500	3680100	3585500
pma3101	Nicotinate D-ribonucleoside	Others	3400300	3749000	3251600	3257700
mws1337	D-Pantothenic Acid	Others	3303400	3261000	3468100	3326600
pmp001273	LysoPC 18:2	Lipids	3278000	3338900	3250000	3204800
pmb2922	Uridine 5'-diphospho-D-glucose	Nucleotides and derivatives	2550800	3298300	3239000	3596000
pme2914	3-Hydroxy-3-methylpentane-1,5-dioic acid	Amino acids and derivatives	3063800	2683800	3260800	2918000
pme3011	γ -Aminobutyric acid*	Organic acids	2725700	3100100	2843500	2793400
mws0282	L-Tryptophan	Amino acids and derivatives	3262200	2855000	2736300	2622900
Zmhn001414	Resveratrol-O-diglucoside	Others	2069600	2352500	2587900	2720100
pmn001419	1-O-[(E)-p-Cumaroyl]- β -D-glucopyranose	Phenolic acids	2541300	2610800	2544500	2364000
pme0040	Adenine	Nucleotides and derivatives	2551900	2629800	2633900	2579100
pmb1650	Octadeca-11E,13E,15Z-trienoic acid	Lipids	2660400	2518300	2757500	2551100
pmn001420	1-O-[(E)-Caffeoyl]- β -D-glucopyranose	Phenolic acids	2280500	2739600	2369600	2326700
pme3017	2-Aminoisobutyric acid*	Amino acids and derivatives	2194500	2547700	2024200	2147200
pme0014	L-Glutamic acid	Amino acids and derivatives	2431700	2439800	2402000	2477500
Rfmb319	Pipecolic acid	Amino acids and derivatives	2148800	2116600	2304000	2133500
pme0193	L-Glutamine	Amino acids and derivatives	2037200	2093300	1939000	1963300
mws5040	Turanose	Others	2194200	2436400	2223200	2549700
pme1178	Guanosine	Nucleotides and derivatives	2211500	2012600	1952200	2127000
pmb3081	Glucarate O-Phosphoric acid	Others	1654400	1986100	2079500	1780100
pme0026	L-(+)-Lysine	Amino acids and derivatives	1962700	1904700	1960300	1871000

pme3184	2'-Deoxyadenosine-5'-monophosphate	Nucleotides and derivatives	1985700	1870200	1929100	1883700
mws0260	L-(+)-Arginine	Amino acids and derivatives	1860900	1920000	1807400	1702600
pme3033	N,N-Dimethylglycine	Amino acids and derivatives	1608900	1877300	1538800	1584100
pme3096	Aminomalonic acid	Organic acids	1242800	1363100	1396100	1278500
mws0219	L-AsparticAcid	Amino acids and derivatives	1371900	1337800	1263200	1376100
pmp001086	5-Hydroxymethylfurfural	Phenolic acids	1343400	1419100	1248700	1487200
mws0275	L-(-)-Malic acid	Organic acids	1305800	1173000	1471900	1401300
Lmhp010190	LysoPC 18:1(2n isomer)	Lipids	1273700	1273200	1140600	1157500
mws0866	D-Glucose 6-phosphate	Others	1252000	1227600	1149100	1104600
mws0628	4-Hydroxybenzaldehyde	Phenolic acids	870570	993660	1109200	1218700
pme0534	Gluconic acid	Others	1104000	1065900	1060900	1047400
mws0983	N-Oleoylethanolamine	Others	1359200	1256600	1253900	1212800
pme3163	D-Sedoheptuiose 7-phosphate	Others	727360	957520	1060200	1067800
pmc0066	2'-Deoxyinosine-5'-monophosphate	Nucleotides and derivatives	1138200	1047600	987930	1027300
mws0227	L-Leucine*	Amino acids and derivatives	859220	906070	872890	883040
pmn001367	Protocatechuic acid-4-glucoside	Phenolic acids	885970	849200	844620	811660
pme1210	L-Methionine	Amino acids and derivatives	820960	934510	868500	817100
mws0671	L-Homoserine*	Organic acids	789980	880470	709560	765430
mws0567	4-Guanidinobutyric acid	Organic acids	809080	900640	878440	883690
pme0516	Inositol	Others	826230	814610	747350	800450
pme1436	p-Coumaric acid	Phenolic acids	960880	855320	781190	733090
Zmxn001997	Isosalicylic acid O-glycoside	Phenolic acids	784500	815190	822150	777900
pme2693	N-Acetylputrescine	Alkaloids	795740	856620	864460	821210
pmb2826	Citramalate	Organic acids	654660	630080	711450	569160
pme3337	N6-Succinyl Adenosine	Nucleotides and derivatives	893620	833670	874440	899690

pmb0501	Agmatine	Alkaloids	770130	873110	821830	801930
pmn001517	3,4,5-Trimethoxyphenyl- β -D-Glucopyranoside	Phenolic acids	784170	682210	800280	704950
pme2266	Biotin	Others	704680	792700	725780	752160
pme3705	D-Glucoronic acid	Others	769020	793410	766830	771100
pmb0981	Adenosine 5'-monophosphate	Nucleotides and derivatives	789390	715590	686450	616950
mws0237	Anchoic Acid	Organic acids	645440	706180	695860	674110
Lmtn002565	1'-O-Vanilloyl- β -D-glucoside	Phenolic acids	587180	691540	723960	698090
HmLn000873	2-O-Galloyl- β -D-glucose	Tannins	666640	753710	689140	754230
pmp001281	LysoPC 18:1	Lipids	608820	643440	565460	591070
Lmtn002233	Androsin	Others	656860	606100	545780	502020
pme3732	Cytidine	Nucleotides and derivatives	567940	627280	587980	587520
mws0232	Riboflavin	Others	631280	614450	612050	674610
pme0281	Terephthalic acid	Phenolic acids	528260	575860	537090	494740
pmb0865	LysoPC 18:3(2n isomer)	Lipids	550250	575960	483950	489330
pme0274	6-Aminocaproic acid	Organic acids	595590	527220	534820	572950
pmb0764	4-Methyl-5-thiazoleethanol	Others	476970	497310	502210	497690
mws0752	Undecylic Acid	Lipids	462960	507140	455210	441700
mws0749	4-Hydroxybenzoic acid	Phenolic acids	435430	478240	516870	502150
mws5038	Isomaltulose*	Others	508700	670850	429320	318440
pme0519	D-(+)-Sucrose*	Others	593290	511120	565550	461930
pmn001706	2-Hydroxyoleanolic acid	Terpenoids	535340	586900	467880	435810
pme2746	Flavin adenine dinucleotide(FAD)	Nucleotides and derivatives	426950	424710	401840	388420
pmp001285	Phthalic anhydride	Phenolic acids	446060	443790	441400	461220
pme1109	Guanine	Nucleotides and derivatives	437760	407990	424380	410620

mws0376	Fumaric acid	Organic acids	455670	381580	408810	371660
mws4170	D-Glucose	Others	423260	476360	431330	410900
pme3313	D-Fructose 6-phosphate	Others	442020	343640	310060	374110
mws1050	O-Acetylserine	Amino acids and derivatives	321770	365650	391270	416660
pme1975	Malonic acid	Organic acids	437630	313350	370410	464500
pmb0874	LysoPE 18:2(2n isomer)	Lipids	359490	341210	319140	308320
mws1038	(R)-Pantetheine	Others	346540	362720	362510	272400
mws0230	L-(-)-Threonine*	Amino acids and derivatives	309740	414150	302700	289660
pmb0530	Nicotinic acid adenine dinucleotide	Nucleotides and derivatives	315050	305900	368430	508090
mws4134	Oxidized Glutathione	Amino acids and derivatives	229470	356080	295960	320640
mws2623	11-Octadecanoic acid(Vaccenic acid)	Lipids	444490	326200	279920	240070
mws0254	L-Histidine	Amino acids and derivatives	327200	257930	295620	285430
pmb3894	Di-O-methylquercetin	Flavonoids	258850	274330	263590	237050
Hmtp000776	4,5,6-Trihydroxy-2-cyclohexen-1-ylideneacetonitrile	Alkaloids	278690	265760	262190	276100
pmb0854	LysoPC 18:3	Lipids	238040	249890	233000	236270
pme2598	3,4-Dihydroxybenzeneacetic acid	Phenolic acids	229940	256800	246580	241350
mws0473	2-Methylsuccinic acid	Organic acids	214180	169270	200510	183650
pmn001694	9,10,13-Trihyroxy-11-octadecadienoic acid	Lipids	225520	239150	227610	208080
mws0133	Nicotinamide	Others	233700	220310	239120	274700
pmb0786	Glucosamine	Others	227960	248700	221120	241940
pmd0132	LysoPC 16:0(2n isomer)	Lipids	171050	217170	217900	261570
pmn001495	Hexadecanoic acid 2,3-dihydroxypropyl ester	Lipids	220700	232670	221240	199430
mws0248	Uridine	Nucleotides and derivatives	221130	226420	223970	203060

Rfmb320	1,2-N-Methylpipecolic acid	Amino acids and derivatives	205540	227060	197100	257050
pme0122	N6-Acetyl-L-lysine	Amino acids and derivatives	162670	242040	213660	221390
mws0258	L-Isoleucine*	Amino acids and derivatives	204050	217330	224580	223450
mws0668	Xanthosine	Nucleotides and derivatives	217270	191840	177610	192420
mws0147	3-Hydroxy-3-methyl butyric acid	Organic acids	195680	199500	186950	199150
Hmtn001302	Glucosyloxybenzoic acid	Phenolic acids	186300	175970	215120	211370
mws1333	Melibiose*	Others	232820	207910	224910	197350
Zmhn002422	Feruloyl glucose	Phenolic acids	183300	202300	194050	181090
Zmhn001926	Salicylic acid O-glycoside	Phenolic acids	167060	200390	190800	216400
pmb2778	9,10-EODE*	Lipids	224020	211180	192120	178690
mws0736	N-Glycyl-L-leucine*	Amino acids and derivatives	188910	201300	187720	232170
pme0490	Nicotinic acid	Others	181410	190090	182990	183150
mws0208	Adipic Acid	Organic acids	165080	177800	175700	171120
pme1014	Menaquinone (K2)	Others	292080	272670	276920	261770
pmb0964	Isopentenyladenine-7-N-glucoside	Nucleotides and derivatives	191300	194320	182550	198410
pmn001511	3-Hydroxy-5-Methylphenol-1-Oxy-β-D-Glucose	Phenolic acids	174290	170680	162300	150850
pmb0449	2-Aminoadipic acid (L-Homoglutamic acid)	Amino acids and derivatives	146430	155030	160340	164860
mws5041	Glycylisoleucine*	Amino acids and derivatives	162420	145440	148390	167680
pmb3099	Diethyl phosphate	Organic acids	115180	122550	112660	121530
pme3083	2-(Formylamino)benzoic acid	Phenolic acids	125740	137560	130060	136830
pme3967	2-(Dimethylamino)guanosine	Nucleotides and derivatives	151930	148340	156820	153050
mws0609	Guanosine 3',5'-cyclic monophosphate	Nucleotides and derivatives	127870	134110	130180	139400
pmb1912	10-Formyl-THF	Alkaloids	140420	146630	126230	137960
mws0458	Vanillin	Phenolic acids	104760	115710	146460	169000

pme0010	L-Serine	Amino acids and derivatives	108860	132020	115680	71245
pme0256	Xanthine	Nucleotides and derivatives	174970	224000	106090	189650
pmb2497	4-Hydroxy-3-methoxymandelate	Phenolic acids	150940	139950	103850	135950
pme1216	2-Picolinic acid	Organic acids	139760	106480	120750	128230
pme0253	N-Acetyl-L-leucine	Amino acids and derivatives	120790	128720	130270	130890
pmb0464	Aspartic acid di-O-glucoside	Amino acids and derivatives	120200	103700	113910	113840
pmb0876	LysoPE 16:0	Lipids	104480	113590	110400	112940
pme3961	Deoxyadenosine	Nucleotides and derivatives	115110	111700	110220	125520
pmb3107	Syringic acid O-glucoside	Phenolic acids	116860	116780	116520	115460
Smsn001839	Dihydrocornin	Terpenoids	123980	102550	114030	111570
mws0126	LysoPC 18:0	Lipids	110730	127610	104170	99573
pme2735	S-Adenosylmethionine	Amino acids and derivatives	158310	106150	150550	131490
mws0675	β-Nicotinamide mononucleotide	Nucleotides and derivatives	120610	130840	82364	110250
pme0183	2-Hydroxy-6-aminopurine	Nucleotides and derivatives	103280	108150	103520	98839
pme2755	N-Acetyl-D-glucosamine	Others	103210	104390	96820	118680
pme2651	NADP (Nicotinamide adenine dinucleotide phosphate)	Nucleotides and derivatives	104870	116530	87845	116930
mws1080	Galactinol*	Others	102460	103680	102570	102470
pmp001276	2,3-Dihydroxypropyl-9,12,15-octadecatrienoate-hexose-hexose	Lipids	135010	130050	88776	97237
pmf0440	4-MethoxycinnaMaldehyde	Phenolic acids	82088	85144	91270	90943
mws5042	Glycylphenylalanine	Amino acids and derivatives	95378	96004	98530	98539
mws0255	Cytosine	Nucleotides and derivatives	84550	100290	81725	85812
mws1060	9-(β-D-Arabinofuranosyl)hypoxanthine	Nucleotides and derivatives	89013	99858	73323	89996

pmn001690	3-Hydroxy-4-isopropylbenzylalcohol 3-glucoside	Phenolic acids	80479	85972	88136	91916
pme2559	N-Acetylaspartate	Amino acids and derivatives	78370	72032	92191	72955
mws2212	Caffeic acid	Phenolic acids	61433	102040	90171	86212
pme0001	Hesperetin 7-O-neohesperidoside(Neohesperidin)	Flavonoids	76665	100180	96232	85678
mws0179	Chlorogenic acid methyl ester	Phenolic acids	101990	85477	62970	73487
Lmhp011562	1-α-Linolenoyl-glycerol*	Lipids	89181	88435	94727	99769
mws0884	Cyclic AMP	Nucleotides and derivatives	76236	69533	76803	70669
pmb0889	Punicic acid	Lipids	84421	78518	85893	84975
Rfmb090	13-Hydroxy-9,11-octadecadienoic acid*	Lipids	85859	82041	77784	77025
mws1491	Linoleic acid	Lipids	54050	67211	72288	70639
pmb2561	N-Acetylmethionine	Amino acids and derivatives	70873	77713	72589	76673
mws0972	5-Hydroxyhexanoic acid	Organic acids	45426	73614	84529	72049
pme0075	N-Acetyl-L-glutamic acid	Amino acids and derivatives	77956	83553	70837	66110
Lmhp012042	2-Linoleoylglycerol*	Lipids	100250	79206	81529	76038
pme1419	L-Methionine methyl ester	Amino acids and derivatives	70425	64512	79340	73598
pmn001688	9S-Hyroxy-10E,12E-octadecadienoic acid*	Lipids	73804	76486	69360	67333
mws0359	Pentadecanoic Acid	Lipids	69228	69105	66762	63808
Lmhp011388	2-γ-Linolenoyl-glycerol*	Lipids	98060	76373	86969	86534
pma0149	Sinapoyl malate	Phenolic acids	61745	65230	65867	73211
mws0520	N-Acetyl-L-tyrosine	Amino acids and derivatives	61266	73822	63439	67035
pmb0374	Aminopurine	Alkaloids	54043	62940	58130	63755
mws0191	Betaine	Alkaloids	57905	74176	57096	54936
pme3186	DL-Glyceraldehyde 3-phosphate	Organic acids	66108	41843	55632	51738

pme0295	4-Acetamidobutyric acid	Organic acids	58392	63377	68182	69483
mws5037	Alanylleucine	Amino acids and derivatives	72036	63836	61222	56059
pme1002	L-Tyramine	Amino acids and derivatives	57833	57771	59800	55936
Lmhp112042	1-Linoleoylglycerol*	Lipids	71383	67175	63257	74151
pme2117	Adenosine 5'-Diphosphate	Nucleotides and derivatives	57864	58288	55472	39326
mws0847	1-Methyladenine	Nucleotides and derivatives	54767	47716	45565	52184
mws0146	Nicotinic Acid Methyl Ester(Methyl Nicotinate)	Alkaloids	37362	49424	52119	65892
mws0001	L-Asparagine	Amino acids and derivatives	37497	70630	37773	36299
mws1200	Trans-4-Hydroxycinnamic Acid Methyl Ester	Phenolic acids	29987	48567	60603	69560
mws0341	(S)-(-)-2-Hydroxyisocaproic acid	Organic acids	43716	48116	49037	46102
pme3388	H-HomoArg-OH	Amino acids and derivatives	48187	48116	39837	36816
pme1286	S-(5'-Adenosy)-L-homocysteine	Amino acids and derivatives	61082	50169	53682	47891
pme2529	1,5-Anhydro-D-glucitol	Others	52694	48111	49578	40710
pmf0359	Skimmin	Lignans and Coumarins	41958	39864	48283	44385
pmb0881	LysoPE 18:2	Lipids	39405	43241	41888	44395
pme0170	N-α-Acetyl-L-arginine	Amino acids and derivatives	32313	42561	39561	51763
pme0278	2,6-Diaminooimelic acid	Amino acids and derivatives	40173	43126	46960	43485
pmb2406	LysoPC 17:0	Lipids	40143	39720	42511	39364
Lmtn002796	Aromadendrin 7-glucoside	Others	40590	43759	50861	30561
pme2596	4-Pyridoxic acid	Others	45212	41393	42439	39325
mws0629	Asp-phe	Amino acids and derivatives	40211	43194	42899	41461
pmn001606	Eicosenoic acid	Lipids	36780	38174	39999	43972
pme3382	N-Acetylthreonine	Amino acids and derivatives	40525	48098	40821	60294

Lmyn000239	Cordycepic acid*	Others	39742	48894	32811	42772
mws0193	L-Homocitrulline	Amino acids and derivatives	42030	37542	43107	46494
pme1184	Deoxyguanosine	Nucleotides and derivatives	35538	40083	37888	42568
pmp001275	3-Hydroxypropyl palmitate glc-glucosamine	Alkaloids	46025	38143	37388	52459
pmn001668	Apigenin-3-O- α -L-rhamnoside	Flavonoids	36927	37170	32592	31428
Zmhn002227	Sinapic acid-glycoside	Phenolic acids	33338	30026	36370	30944
mws4176	Alanylphenylalanine	Amino acids and derivatives	38618	34518	35971	35059
pmp001282	Propyl2-(trimethylammonio)ethyl phosphate	Others	52562	42606	22010	27752
pmb0197	N2-methylguanosine	Nucleotides and derivatives	26719	41040	29935	36029
Lmdp003090	Dihydroquercetin(Taxifolin)	Flavonoids	31805	34583	36493	35482
Lmhn002683	p-Coumaroylcaffeoyltartaric acid	Phenolic acids	35287	34308	29325	29044
pme1194	Deoxycytidine	Nucleotides and derivatives	27195	31773	29751	28147
pme3174	Cytidine 5'-monophosphate(Cytidylic acid)	Nucleotides and derivatives	24792	29550	28033	34545
YC512118	Octadecenoic amide	Others	12817	9371.2	10577	9276
pme0264	Thymidine	Nucleotides and derivatives	30142	30108	28073	29991
mws1499	D-(-)-Arabinose	Others	31285	31540	31350	32270
pmb2792	13-HOTrE(r)	Lipids	22613	20995	17542	16479
pmb0856	LysoPE 18:1(2n isomer)	Lipids	28018	29814	26739	27853
pmb2363	MAG(18:1)	Lipids	33227	27923	27223	30095
pme1021	D-(+)-Glucono-1,5-lactone	Others	23806	33946	24436	32671
pmb2653	D(+)-Melezitose O-rhamnoside	Others	20959	24885	40075	23794
pme3968	7-Methylguanine	Nucleotides and derivatives	26167	26310	29916	28193
Lmhp008589	LysoPE 18:3(2n isomer)	Lipids	25158	25044	24341	25371
mws0177	2-Furanoic acid	Organic acids	23366	20172	23939	29736
pmn001336	Aloeemodin-8-O-D-glucopyranoside	Quinones	26140	25976	23777	25389

pme0195	L-Cysteine	Amino acids and derivatives	30483	20963	23964	23278
pmn001352	6-Hydroxyrumicin-8-O-D-glucopyranoside	Quinones	36693	25712	16958	17399
Lmdp003994	Wistin (6,4'-Dimethoxyisoflavone-7-glucoside)	Flavonoids	24811	25709	23000	24135
pmb2654	Anthranilate O-hexosyl-O-hexoside	Phenolic acids	22886	21564	27858	26095
pme0241	Benzoic acid	Phenolic acids	32750	28652	27448	25654
mws0011	Syringin	Phenolic acids	35851	22951	15559	13088
Lmtn003598	3-Prenyl-4-O-β-D-glucopyranosyloxy-4-hydroxyl-benzoic acid	Phenolic acids	20980	25753	21512	20428
pmb0801	4-Pyridoxic acid O-hexoside	Others	23330	22735	20319	21613
Hmpp003242	Isorhamnetin-3-O-β-D-glucoside	Flavonoids	22690	22142	21888	20862
pme2527	L-Ornithine	Amino acids and derivatives	21367	19874	18276	19722
pmp001270	LysoPC 16:1	Lipids	18048	18787	14949	15005
pmn001681	1-(4-Methoxyphenyl)-1-propanol	Phenolic acids	18033	25691	26770	32271
Lmhn002926	p-Coumaroylmalic acid	Phenolic acids	15913	17174	14984	12603
Lmhp009590	LysoPC 17:1	Lipids	20004	18969	16594	16430
pmp001312	6-Hydroxykaempferol-3,7,6-O-triglycoside	Flavonoids	19275	20628	20516	19060
pme0008	L-Citrulline	Amino acids and derivatives	17297	21058	16897	22058
YC512119	Phytosphingosine	Others	17676	20391	16235	18732
mws0242	SubericAcid	Organic acids	14429	15467	13218	13309
mws1421	α-Viniferin	Others	12785	12207	12247	11689
pme0181	1-Methylhistidine	Amino acids and derivatives	16648	23803	13302	13185
Lmhn003074	Feruloylmalic acid	Phenolic acids	19994	14921	8876.3	8497.6
pme1730	D-Erythronolactone	Organic acids	17357	13355	12171	19687
mws1383	Lumichrome	Alkaloids	14789	15782	15454	15125

mws2523	Trehalose 6-phosphate	Others	12390	14029	11984	13272
mws0852	Methotrexate	Nucleotides and derivatives	13771	14414	14485	14857
pmb2260	LysoPC 15:1	Lipids	15007	12626	12454	11118
pme0033	Hypoxanthine	Nucleotides and derivatives	15361	13675	13677	13940
mws0124	N-(3-Indolylacetyl)-L-alanine	Amino acids and derivatives	16175	13079	11725	11762
pmb2804	13-HPODE	Lipids	14400	13216	12674	11912
mws0361	Palmitoleic Acid	Lipids	11988	12567	12360	13811
mws0572	5-Methylcytosine	Nucleotides and derivatives	6709.4	12328	10671	10621
pme0120	5-Aminovaleric acid*	Amino acids and derivatives	10344	14129	11718	13666
pme1738	3-Carbamyl-1-methylpyridinium(1-Methylnicotinamide)	Others	10213	13436	14504	13899
pmb2657	Argininosuccinic acid	Organic acids	12909	12271	10006	11222
mws0981	Isoxanthopterin	Nucleotides and derivatives	10354	12767	11306	9463.4
pmd0160	LysoPE 16:0(2n isomer)	Lipids	9103.6	10258	10736	11903
pme1187	5-Methyluridine	Nucleotides and derivatives	9797.8	11068	11713	11369
mws1589	Panose*	Others	11786	9206.2	12962	8884.2
Hmlp001371	Cyclo(Tyr-Ala)	Amino acids and derivatives	10104	8988	9531.4	13431
pmb2857	L-Glutamic acid O-glycoside	Amino acids and derivatives	7790.3	10074	9699.6	10576
mws5035	Leucylphenylalanine	Amino acids and derivatives	12355	10412	10486	11792
pmb3075	3-O-p-Coumaroylshikimic acid	Phenolic acids	10005	10166	12334	10361
Lmhp008718	LysoPC 17:2	Lipids	9548.2	7985.7	8295.4	9287.7
mws0057	Eriodictyol 7-O-glucoside	Flavonoids	8302.8	9447.3	8062.8	8448.1
pmb2799	12,13-EODE	Lipids	8907.2	10366	9768.9	8934.9
Hmjn008136	Camaldulenic acid	Terpenoids	12508	12400	11071	10333
pmb0962	Lysine butyrate	Amino acids and derivatives	8319.9	7004.5	13560	11288

mws1078	Anthranilic Acid	Phenolic acids	9710.3	11382	7578.1	7879.7
pmb1096	Indole	Alkaloids	9133.2	8909.1	8483.7	10454
mws2218	Caffeine	Alkaloids	9321.4	9376.7	9455.3	9612.7
mws1420	ε-Viniferin	Others	9621.1	9610.6	7481.3	5678.7
pmd0136	LysoPC 18:0(2n isomer)	Lipids	6421.6	8590	7418.7	8027.4
pme2237	Dulcitol*	Others	6441.6	7403.2	5765.4	7933.5
pmf0297	1-Eicosanol	Lipids	7647.7	6902.4	7189.9	6394.9
Hmbn002174	4-Hydroxyacetophenone	Phenolic acids	6911.3	6928.3	7192.6	9094
Hmcp002268	Limocitrin 3-rhamnoside	Flavonoids	11896	10468	8230.5	3473.1
mws0596	3-Hydroxyanthranilic acid	Organic acids	9454.4	9215.3	6957.5	10376
pmb2791	9-HpOTrE	Lipids	6465.4	6649.8	6208.1	6965.9
mws1212	Methyl ferulate	Phenolic acids	4351.6	5820.4	6928.2	5630.2
pmb2640	Lauric acid	Lipids	5635.2	5929.1	5691	5401.4
mws0289	LysoPE 18:1	Lipids	6352.7	6625.8	7045.9	8492.1
pme0500	D-(+)-Melezitose*	Others	6224.3	5065.3	3389.4	7445.8
pmn001691	9,12,13-Trihyroxy-10,15-octadecadienoic acid	Lipids	5384	5891.6	5015.7	5134.4
pmn001695	Trihydroxycinnamoylquinic acid	Phenolic acids	7049.6	8388.9	7122.2	6560
mws0120	Choline alfoscerate	Lipids	4127.7	5166.1	4404.4	3292.8
Lmhp008801	LysoPE 18:3	Lipids	4813.5	5430.2	4481.8	5571
pme2890	L-Homocystine	Amino acids and derivatives	5789.6	6120.6	4489.4	7727
Lmhp009129	LysoPC 15:0	Lipids	3170.4	4035.4	5532	6333.5
mws0636	Phe-Phe	Amino acids and derivatives	6047.4	4451.8	5266.1	4784.1
pmb2786	9-HOTrE	Lipids	5061.7	5290.4	4620.5	4047.3
pmb1283	L-Glutaminyl-L-valyl-L-valyl-L-cysteine	Amino acids and derivatives	4927.3	4168.9	3692	2936.9

(Treatments: C11 and C11E)								
Index	Compounds	Class I	C11_1	C11_2	C11_3	C11E_1	C11E_2	C11E_3
mws0470	Methylmalonic acid*	Organic acids	41938000	45410000	46843000	41550000	46783000	39481000
mws0192	Succinic acid*	Organic acids	39072000	43744000	45414000	40409000	44425000	40740000
pme0230	Adenosine	Nucleotides and derivatives	18874000	18471000	19523000	19584000	21208000	20688000
pme0021	L-Phenylalanine	Amino acids and derivatives	15262000	14680000	15048000	16882000	16171000	16684000
mws0277	Kinic acid	Organic acids	16120000	15902000	14941000	18500000	19542000	18794000
pmb0789	Pyridoxine-5'-O-glucoside	Others	15000000	17113000	16675000	14818000	15501000	16293000
mws0216	Trans-4-Hydroxy-L-proline	Amino acids and derivatives	11413000	9587000	8479900	7807000	12982000	14135000
mws0281	Citric Acid	Organic acids	16949000	16389000	13877000	15162000	15621000	16536000
pmc0274	6-Methylmercaptopurine	Nucleotides and derivatives	10935000	9952500	10418000	11955000	11339000	11627000
Rfmb324	Trigonelline	Alkaloids	9286300	9416900	9525900	10896000	11794000	12382000
pme0006	L-Proline	Amino acids and derivatives	9514700	8205800	9671900	8936400	8893400	9717900
pmn001578	Hexadecanoic acid	Lipids	10164000	10112000	10029000	8530100	8182700	8089900
mws0119	Myristic Acid	Lipids	6967800	7331200	7085500	7127700	7515500	6737600
pmp001287	N-Benzylmethylene isomethylamine	Alkaloids	5012500	4759800	5166800	5961600	5423900	5760600
pme1474	5'-Deoxy-5'-(methylthio)adenosine	Nucleotides and derivatives	4803900	5095200	6090500	7036200	6813500	5967500
mws1489	Stearic Acid	Lipids	8000400	6751000	6900600	5846800	5573000	5618400

mws0256	L-Valine*	Amino acids and derivatives	3759200	3773700	3972500	5557600	5306700	5626600
Zmhn001970	Piceid	Others	5190700	6331200	7433800	2815100	3411400	2759100
pmb0855	LysoPC 16:0	Lipids	6941000	3904500	4921700	5588800	5233600	6325400
mws0250	L-(-)-Tyrosine	Amino acids and derivatives	4581400	4241700	5371700	4824100	4327900	4191300
pme1383	Pyridoxine	Others	5769800	6931100	6288300	3545500	3702900	3982000
mws0366	γ-Linolenic Acid	Lipids	4609900	6427100	4932200	4535100	4668700	4648900
pmp001198	6-Deoxyfagomine	Alkaloids	2660500	2626800	3016100	3904600	3639900	3914500
pmb3101	2-Isopropylmalate	Organic acids	2266300	3696300	3041600	4168100	4378300	4180400
Hmsn000210	Methyl 7,10-hexadecadienoate	Lipids	3741300	3813000	3379700	3348700	3862600	3453600
pme2380	A-Ketoglutaric acid	Organic acids	4757500	4836700	4271600	3608800	3509500	3484100
pma3101	Nicotinate D-ribonucleoside	Others	2781900	2628800	2663600	3754300	4118500	4212000
mws1337	D-Pantothenic Acid	Others	2901800	3504900	3379200	3388800	3096000	3349600
pmp001273	LysoPC 18:2	Lipids	4272300	2368900	2747700	2935400	2977400	3620200
pmb2922	Uridine 5'-diphospho-D-glucose	Nucleotides and derivatives	2503000	2343800	2369000	3126200	3209700	2976400
pme2914	3-Hydroxy-3-methylpentane-1,5-dioic acid	Amino acids and derivatives	3431100	3219900	3787100	2756200	2906800	2222700
pme3011	γ-Aminobutyric acid*	Organic acids	2480600	2533500	2331400	2688000	2858500	2923900
mws0282	L-Tryptophan	Amino acids and derivatives	2429000	1899000	2487000	1881900	1763000	1752900
Zmhn001414	Resveratrol-O-diglucoside	Others	1896300	1829700	2098000	3886200	4133000	3792700
pmn001419	1-O-[(E)-p-Cumaroyl]-β-D-glucopyranose	Phenolic acids	1356100	1235100	1897000	2093700	2382400	2429800

pme0040	Adenine	Nucleotides and derivatives	2529500	2482000	2471100	2787200	2915500	2882100
pmb1650	Octadeca-11E,13E,15Z-trienoic acid	Lipids	2653000	2187000	2423300	3810700	2636400	1941800
pmn001420	1-O-[(E)-Caffeoyl]- β -D-glucopyranose	Phenolic acids	2754100	2704200	2758100	2275200	1912800	1905000
pme3017	2-Aminoisobutyric acid*	Amino acids and derivatives	2395100	2408100	2246400	2407300	2610800	2549100
pme0014	L-Glutamic acid	Amino acids and derivatives	2529900	2345200	1911200	1868900	1620300	1709500
Rfmb319	Pipecolic acid	Amino acids and derivatives	2726900	2741200	2382200	2165400	2233900	1817300
pme0193	L-Glutamine	Amino acids and derivatives	1642700	1457500	1595300	1872600	1691200	1923400
mws5040	Turanose	Others	1966400	1495000	1644300	2017600	1693800	1772700
pme1178	Guanosine	Nucleotides and derivatives	1709700	1659600	1592000	1902100	1985600	2530900
pmb3081	Glucarate O-Phosphoric acid	Others	1873800	1781900	2269500	1826100	2144500	2049700
pme0026	L-(+)-Lysine	Amino acids and derivatives	1544400	1313100	1522500	1726100	1555500	1876900
pme3184	2'-Deoxyadenosine-5'-monophosphate	Nucleotides and derivatives	1706800	1874200	2169500	1821400	1824900	1916000
mws0260	L-(+)-Arginine	Amino acids and derivatives	1604500	1220200	1324800	1617000	1305000	1504700
pme3033	N,N-Dimethylglycine	Amino acids and derivatives	1445900	1283600	1397500	1673300	1924700	1900300

pme3096	Aminomalonic acid	Organic acids	1613300	1795800	1870900	1663900	1682000	1639000
mws0219	L-AsparticAcid	Amino acids and derivatives	925950	970080	928340	1028200	1020900	1020800
pmp001086	5-Hydroxymethylfurfural	Phenolic acids	1321600	1404600	1168800	1382700	1398800	1365700
mws0275	L-(-)-Malic acid	Organic acids	1329700	1452100	1234100	1172400	1425800	1289000
Lmhp010190	LysoPC 18:1(2n isomer)	Lipids	1934600	1046900	1235100	1141600	1031000	1349600
mws0866	D-Glucose 6-phosphate	Others	1089900	1217700	1273200	1197200	1144100	1302700
mws0628	4-Hydroxybenzaldehyde	Phenolic acids	764580	694750	832360	1402400	1299400	1246900
pme0534	Gluconic acid	Others	793980	804920	756900	1503000	1598200	1494200
mws0983	N-Oleoylethanolamine	Others	1169000	1305100	1190100	753770	708910	807740
pme3163	D-Sedoheptuiose 7-phosphate	Others	891660	822320	964520	1022100	969160	946430
pmc0066	2'-Deoxyinosine-5'-monophosphate	Nucleotides and derivatives	1277400	1247000	1140700	874240	947260	920670
mws0227	L-Leucine*	Amino acids and derivatives	558540	551800	644450	815120	806210	762440
pmn001367	Protocatechuic acid-4-glucoside	Phenolic acids	1104500	1064100	1018600	1099900	1026300	755150
pme1210	L-Methionine	Amino acids and derivatives	571550	523330	645930	821130	801750	797060
mws0671	L-Homoserine*	Organic acids	648900	527340	544440	720900	682500	758980
mws0567	4-Guanidinobutyric acid	Organic acids	598690	583580	662260	855970	903740	924980
pme0516	Inositol	Others	818440	831940	890510	797370	736190	830540
pme1436	p-Coumaric acid	Phenolic acids	1179100	1067400	1215300	850210	691830	781980
Zmxn001997	Isosalicylic acid O-glycoside	Phenolic acids	872980	763980	984610	588780	592480	575740
pme2693	N-Acetylputrescine	Alkaloids	1104400	1258700	1281300	720120	856600	913060
pmb2826	Citramalate	Organic acids	533680	793120	813870	1060100	1057700	1003300

pme3337	N6-Succinyl Adenosine	Nucleotides and derivatives	692380	704400	700440	909410	821910	820800
pmb0501	Agmatine	Alkaloids	1082500	1176800	1211100	739730	807380	842840
pmn001517	3,4,5-Trimethoxyphenyl- β -D-Glucopyranoside	Phenolic acids	590570	552290	569720	825480	764500	605610
pme2266	Biotin	Others	419050	502020	540240	826980	790380	912940
pme3705	D-Glucoronic acid	Others	304400	321470	318580	1036900	1128000	1080700
pmb0981	Adenosine 5'-monophosphate	Nucleotides and derivatives	609850	803950	774260	755040	859610	823230
mws0237	Anchoic Acid	Organic acids	634430	667250	702340	744050	729290	778440
Lmtn002565	1'-O-Vanilloyl- β -D-glucoside	Phenolic acids	315360	555180	455690	735570	914710	800940
HmLn000873	2-O-Galloyl- β -D-glucose	Tannins	635940	970830	962400	208090	344600	219940
pmp001281	LysoPC 18:1	Lipids	909100	432830	534860	524560	487110	651810
Lmtn002233	Androsin	Others	996940	748410	809300	565830	500090	483780
pme3732	Cytidine	Nucleotides and derivatives	486870	434440	427770	582990	707790	930430
mws0232	Riboflavin	Others	491970	498310	481220	567030	576390	579660
pme0281	Terephthalic acid	Phenolic acids	533680	591170	616500	581430	628180	623980
pmb0865	LysoPC 18:3(2n isomer)	Lipids	536660	379070	432960	482700	489330	714920
pme0274	6-Aminocaproic acid	Organic acids	400270	310790	163560	325280	454370	473610
pmb0764	4-Methyl-5-thiazoleethanol	Others	335610	492050	653940	625550	668690	706910
mws0752	Undecylic Acid	Lipids	480240	517960	558080	528040	487810	427170
mws0749	4-Hydroxybenzoic acid	Phenolic acids	495990	469020	618310	511900	501400	482590
mws5038	Isomaltulose*	Others	970660	555350	494360	496090	391420	379660
pme0519	D-(+)-Sucrose*	Others	583240	720030	454790	331400	511520	445200

pmn001706	2-Hydroxyoleanolic acid	Terpenoids	79351	112540	122950	2207900	2019000	1264100
pme2746	Flavin adenine dinucleotide(FAD)	Nucleotides and derivatives	373480	471900	415200	491850	473520	460670
pmp001285	Phthalic anhydride	Phenolic acids	436360	400290	415980	645680	451450	327280
pme1109	Guanine	Nucleotides and derivatives	462340	322820	487700	511260	505500	484560
mws0376	Fumaric acid	Organic acids	500200	466380	479390	468090	436670	295380
mws4170	D-Glucose	Others	572030	449790	407760	317700	320870	332470
pme3313	D-Fructose 6-phosphate	Others	533130	522960	588120	423700	312920	264150
mws1050	O-Acetylserine	Amino acids and derivatives	253580	295670	391160	576100	552150	681810
pme1975	Malonic acid	Organic acids	382920	387210	313390	293240	324360	346150
pmb0874	LysoPE 18:2(2n isomer)	Lipids	456760	193740	208570	283310	262390	360710
mws1038	(R)-Pantetheine	Others	324080	362680	324080	348040	444420	349530
mws0230	L-(-)-Threonine*	Amino acids and derivatives	243300	236250	221950	432510	262960	289450
pmb0530	Nicotinic acid adenine dinucleotide	Nucleotides and derivatives	253630	327630	322540	353580	331090	274010
mws4134	Oxidized Glutathione	Amino acids and derivatives	237220	215380	193950	248070	270370	263970
mws2623	11-Octadecanoic acid(Vaccenic acid)	Lipids	587430	676940	548180	203170	212360	173230
mws0254	L-Histidine	Amino acids and derivatives	269270	202410	231240	198560	178560	206050
pmb3894	Di-O-methylquercetin	Flavonoids	396570	300100	355860	229160	231870	246250

Hmtp000776	4,5,6-Trihydroxy-2-cyclohexen-1-ylideneacetonitrile	Alkaloids	190710	194870	214300	234980	216820	216770
pmb0854	LysoPC 18:3	Lipids	285450	185380	209560	249560	234050	314620
pme2598	3,4-Dihydroxybenzeneacetic acid	Phenolic acids	373880	353750	303130	256780	263590	190980
mws0473	2-Methylsuccinic acid	Organic acids	204700	285620	258200	227330	243950	158550
pmn001694	9,10,13-Trihyroxy-11-octadecadienoic acid	Lipids	340520	270520	317860	205610	202720	219900
mws0133	Nicotinamide	Others	124820	130620	275400	409050	379140	367770
pmb0786	Glucosamine	Others	225050	217480	211120	214530	230920	239420
pmd0132	LysoPC 16:0(2n isomer)	Lipids	205330	140100	177310	211640	212980	252660
pmn001495	Hexadecanoic acid 2,3-dihydroxypropyl ester	Lipids	341220	261890	311200	196070	192940	199170
mws0248	Uridine	Nucleotides and derivatives	219860	191540	173400	256290	265970	301330
Rfmb320	1,2-N-Methylpipecolic acid	Amino acids and derivatives	166730	172510	138370	147810	179740	263470
pme0122	N6-Acetyl-L-lysine	Amino acids and derivatives	211020	148630	253170	186660	220170	199300
mws0258	L-Isoleucine*	Amino acids and derivatives	129940	125770	149570	177610	170690	195530
mws0668	Xanthosine	Nucleotides and derivatives	221660	229440	225180	160530	172630	197410
mws0147	3-Hydroxy-3-methyl butyric acid	Organic acids	212870	224720	231260	160000	151280	136700
Hmtn001302	Glucosyloxybenzoic acid	Phenolic acids	163210	172360	175670	216470	280680	190850
mws1333	Melibiose*	Others	334200	196930	243680	173930	140800	175150
Zmhn002422	Feruloyl glucose	Phenolic acids	70909	82370	90710	287560	189140	175630

Zmhn001926	Salicylic acid O-glycoside	Phenolic acids	174990	158120	173940	207590	288380	163690
pmb2778	9,10-EODE*	Lipids	186950	246020	183520	137700	148940	145730
mws0736	N-Glycyl-L-leucine*	Amino acids and derivatives	206130	158770	192300	151170	175510	169130
pme0490	Nicotinic acid	Others	157150	157530	195800	242130	240470	279870
mws0208	Adipic Acid	Organic acids	157070	165570	184620	171950	161440	183990
pme1014	Menaquinone (K2)	Others	137870	165030	117910	182400	132160	132580
pmb0964	Isopentenyladenine-7-N-glucoside	Nucleotides and derivatives	181590	167810	165360	157520	126530	142620
pmn001511	3-Hydroxy-5-Methylphenol-1-Oxy-β-D-Glucose	Phenolic acids	107800	158500	115660	197520	224190	196310
pmb0449	2-Aminoadipic acid (L-Homoglutamic acid)	Amino acids and derivatives	110960	102390	125510	177640	166080	200170
mws5041	Glycylisoleucine*	Amino acids and derivatives	149270	121150	130170	130460	131670	144500
pmb3099	Diethyl phosphate	Organic acids	194370	201180	226740	137930	156770	160720
pme3083	2-(Formylamino)benzoic acid	Phenolic acids	151200	154400	179130	138610	132060	141750
pme3967	2-(Dimethylamino)guanosine	Nucleotides and derivatives	114880	113990	108340	136760	147730	163530
mws0609	Guanosine 3',5'-cyclic monophosphate	Nucleotides and derivatives	124090	144180	153580	130390	117200	107570
pmb1912	10-Formyl-THF	Alkaloids	116370	130640	110470	148580	131700	138850
mws0458	Vanillin	Phenolic acids	84177	86398	108070	161480	171650	166650
pme0010	L-Serine	Amino acids and derivatives	162000	176190	130090	121980	110810	79644

pme0256	Xanthine	Nucleotides and derivatives	78980	91852	75982	90591	81608	217440
pmb2497	4-Hydroxy-3-methoxymandelate	Phenolic acids	164150	139860	136980	103640	123730	131410
pme1216	2-Picolinic acid	Organic acids	92566	91664	131680	166960	158760	165740
pme0253	N-Acetyl-L-leucine	Amino acids and derivatives	289130	239510	523940	106710	99463	112640
pmb0464	Aspartic acid di-O-glucoside	Amino acids and derivatives	86877	78944	114100	82767	100890	114180
pmb0876	LysoPE 16:0	Lipids	146640	48091	65445	92565	78829	118490
pme3961	Deoxyadenosine	Nucleotides and derivatives	93745	106760	95068	102810	128890	149840
pmb3107	Syringic acid O-glucoside	Phenolic acids	118560	120790	123410	81656	71566	69821
Smsn001839	Dihydrocornin	Terpenoids	95260	106620	107120	111170	125670	124920
mws0126	LysoPC 18:0	Lipids	159170	53229	64097	74735	73459	94889
pme2735	S-Adenosylmethionine	Amino acids and derivatives	155460	133500	110510	96358	79086	107380
mws0675	β-Nicotinamide mononucleotide	Nucleotides and derivatives	101300	130130	103420	97400	114110	147540
pme0183	2-Hydroxy-6-aminopurine	Nucleotides and derivatives	109430	91517	105300	115010	117240	123410
pme2755	N-Acetyl-D-glucosamine	Others	101840	100520	92212	106630	110900	106860
pme2651	NADP (Nicotinamide adenine dinucleotide phosphate)	Nucleotides and derivatives	120640	116470	123190	100870	103110	100860
mws1080	Galactinol*	Others	99704	84171	80045	42527	52999	68502
pmp001276	2,3-Dihydroxypropyl-9,12,15-octadecatrienoate-hexose-hexose	Lipids	87352	90643	87664	137000	118900	145770

pmf0440	4-MethoxycinnaMaldehyde	Phenolic acids	112810	122590	129150	109510	113340	96223
mws5042	Glycylphenylalanine	Amino acids and derivatives	99041	73828	87940	84925	97190	88706
mws0255	Cytosine	Nucleotides and derivatives	83109	74558	69885	98420	120170	141090
mws1060	9-(β -D-Arabinofuranosyl)hypoxanthine	Nucleotides and derivatives	114890	98842	99617	77339	92756	106940
pmn001690	3-Hydroxy-4-isopropylbenzylalcohol 3-glucoside	Phenolic acids	38292	55439	59971	114520	111000	122780
pme2559	N-Acetylaspartate	Amino acids and derivatives	145360	158820	173670	71199	66594	60452
mws2212	Caffeic acid	Phenolic acids	68878	58066	65037	96283	99223	97504
pme0001	Hesperetin 7-O-neohesperidoside(Neohesperidin)	Flavonoids	9	9	9	5589.9	9	314980
mws0179	Chlorogenic acid methyl ester	Phenolic acids	66819	84665	80293	77642	75195	73494
Lmhp011562	1-α-Linolenoyl-glycerol*	Lipids	73104	114820	77868	64111	68582	76035
mws0884	Cyclic AMP	Nucleotides and derivatives	69056	65400	60500	77806	85841	77361
pmb0889	Punicic acid	Lipids	72501	83309	69961	54021	57373	54647
Rfmb090	13-Hydroxy-9,11-octadecadienoic acid*	Lipids	66543	93795	61548	57343	63265	51619
mws1491	Linoleic acid	Lipids	59498	53875	51414	86661	84877	87451
pmb2561	N-Acetylmethionine	Amino acids and derivatives	61722	61946	53925	79332	103440	69863
mws0972	5-Hydroxyhexanoic acid	Organic acids	29176	43395	39993	78342	76427	63338

pme0075	N-Acetyl-L-glutamic acid	Amino acids and derivatives	146640	128670	142390	66339	56596	58168
Lmhp012042	2-Linoleoylglycerol*	Lipids	69410	104890	66816	68163	58341	70644
pme1419	L-Methionine methyl ester	Amino acids and derivatives	68571	74604	63731	71475	67608	69395
pmn001688	9S-Hyroxy-10E,12E-octadecadienoic acid*	Lipids	57944	83798	57860	50902	59737	48657
mws0359	Pentadecanoic Acid	Lipids	65575	64294	61383	42645	36468	41911
Lmhp011388	2-γ-Linolenoyl-glycerol*	Lipids	52716	100880	59001	57465	61438	81281
pma0149	Sinapoyl malate	Phenolic acids	65803	58910	59734	72445	60326	59074
mws0520	N-Acetyl-L-tyrosine	Amino acids and derivatives	306910	210300	485370	20600	17191	17090
pmb0374	Aminopurine	Alkaloids	54375	51921	54744	63142	58680	63255
mws0191	Betaine	Alkaloids	59288	64110	66597	72238	69924	76107
pme3186	DL-Glyceraldehyde 3-phosphate	Organic acids	53026	57708	51209	59804	70398	48874
pme0295	4-Acetamidobutyric acid	Organic acids	65178	75182	69810	64450	64587	86664
mws5037	Alanylleucine	Amino acids and derivatives	41904	48282	51923	65490	63245	66704
pme1002	L-Tyramine	Amino acids and derivatives	22059	22452	34995	41827	42071	141730
Lmhp112042	1-Linoleoylglycerol*	Lipids	56303	86619	52262	50971	42693	41045
pme2117	Adenosine 5'-Diphosphate	Nucleotides and derivatives	62310	73108	68167	55116	51351	49710
mws0847	1-Methyladenine	Nucleotides and derivatives	41224	43595	40341	52578	54364	58021

mws0146	Nicotinic Acid Methyl Ester(Methyl Nicotinate)	Alkaloids	25166	30466	39287	58305	66931	75713
mws0001	L-Asparagine	Amino acids and derivatives	38045	46624	51430	53291	50028	46068
mws1200	Trans-4-Hydroxycinnamic Acid Methyl Ester	Phenolic acids	50000	45371	49044	46304	48994	46730
mws0341	(S)-(-)-2-Hydroxyisocaproic acid	Organic acids	58539	49191	73516	51615	55086	53967
pme3388	H-HomoArg-OH	Amino acids and derivatives	50046	27808	35510	50805	37978	43095
pme1286	S-(5'-Adenosy)-L-homocysteine	Amino acids and derivatives	39852	36101	47235	46569	56764	57410
pme2529	1,5-Anhydro-D-glucitol	Others	48977	57761	49155	45347	45120	67550
pmf0359	Skimmin	Lignans and Coumarins	79259	92192	84179	39732	37247	27900
pmb0881	LysoPE 18:2	Lipids	57919	14698	20807	34920	33851	50378
pme0170	N-α-Acetyl-L-arginine	Amino acids and derivatives	97011	89812	189140	31124	30106	32724
pme0278	2,6-Diaminooimelic acid	Amino acids and derivatives	41823	26757	39342	48919	41008	38656
pmb2406	LysoPC 17:0	Lipids	58907	26957	31534	36099	34800	43915
Lmtn002796	Aromadendrin 7-glucoside	Others	21962	55749	30416	42375	52181	51623
pme2596	4-Pyridoxic acid	Others	35969	33909	35932	44726	38376	39107
mws0629	Asp-phe	Amino acids and derivatives	37217	31901	43137	38629	45611	42560
pmn001606	Eicosenoic acid	Lipids	36555	55755	43576	36172	45323	40407

pme3382	N-Acetylthreonine	Amino acids and derivatives	69726	57158	76096	32579	32858	40748
Lmyn000239	Cordycepic acid*	Others	41324	49333	41609	30575	42988	37035
mws0193	L-Homocitrulline	Amino acids and derivatives	39605	31602	41312	35276	35798	43571
pme1184	Deoxyguanosine	Nucleotides and derivatives	28648	32189	36979	41803	46159	47368
pmp001275	3-Hydroxypropyl palmitate glc-glucosamine	Alkaloids	24537	28458	24783	49691	48385	56653
pmn001668	Apigenin-3-O-α-L-rhamnoside	Flavonoids	37715	38006	30335	41401	40434	36259
Zmhn002227	Sinapic acid-glycoside	Phenolic acids	26642	30786	29626	45434	46848	34830
mws4176	Alanylphenylalanine	Amino acids and derivatives	34314	28249	35646	26143	36458	33181
pmp001282	Propyl2-(trimethylammonio)ethyl phosphate	Others	77859	36120	38631	22789	22020	24996
pmb0197	N2-methylguanosine	Nucleotides and derivatives	31906	27179	25635	44251	38528	36140
Lmdp003090	Dihydroquercetin(Taxifolin)	Flavonoids	24654	28563	22464	35683	35382	36879
Lmhn002683	p-Coumaroylcaffeoyltartaric acid	Phenolic acids	114060	108980	111030	12322	12651	13976
pme1194	Deoxycytidine	Nucleotides and derivatives	22660	27578	24241	31990	31509	30626
pme3174	Cytidine 5'-monophosphate(Cytidylic acid)	Nucleotides and derivatives	29759	29556	32597	27706	30519	30960
YC512118	Octadecenoic amide	Others	29119	25994	40739	18017	13040	11513
pme0264	Thymidine	Nucleotides and derivatives	28203	27770	27145	28639	32670	35777

mws1499	D-(-)-Arabinose	Others	35268	25206	31516	25065	25887	21000
pmb2792	13-HOTrE(r)	Lipids	18977	21752	22174	32372	19018	22660
pmb0856	LysoPE 18:1(2n isomer)	Lipids	36714	16710	22246	19772	19415	29553
pmb2363	MAG(18:1)	Lipids	23949	22884	17252	35511	32119	40559
pme1021	D-(+)-Glucono-1,5-lactone	Others	29471	24184	36320	33922	31032	23057
pmb2653	D(+)-Melezitose O-rhamnoside	Others	44610	45416	34617	19913	24613	21477
pme3968	7-Methylguanine	Nucleotides and derivatives	22915	26519	28509	32274	31543	32525
Lmhp008589	LysoPE 18:3(2n isomer)	Lipids	32189	12628	15041	17725	17696	36048
mws0177	2-Furanoic acid	Organic acids	34852	40125	54812	40523	38520	34456
pmn001336	Aloeemodin-8-O-D-glucopyranoside	Quinones	20229	13294	21401	23943	23958	21352
pme0195	L-Cysteine	Amino acids and derivatives	13156	23918	25905	22450	20221	26201
pmn001352	6-Hydroxyrumicin-8-O-D-glucopyranoside	Quinones	43964	58469	61917	12293	11587	14923
Lmdp003994	Wistin (6,4'-Dimethoxyisoflavone-7-glucoside)	Flavonoids	17276	23428	18565	36084	40557	39346
pmb2654	Anthranilate O-hexosyl-O-hexoside	Phenolic acids	29416	23715	18100	23029	20061	19505
pme0241	Benzoic acid	Phenolic acids	21214	21510	27323	24515	24666	14161
mws0011	Syringin	Phenolic acids	25215	19591	28582	10392	13722	14613
Lmtn003598	3-Prenyl-4-O-β-D-glucopyranosyloxy-4-hydroxyl-benzoic acid	Phenolic acids	17147	14181	10736	24733	24970	33137
pmb0801	4-Pyridoxic acid O-hexoside	Others	17385	18245	16207	20555	24106	25380

Hmpp003242	Isorhamnetin-3-O-β-D-glucoside	Flavonoids	11430	17886	13317	24241	22811	28281
pme2527	L-Ornithine	Amino acids and derivatives	18715	17194	19073	18009	13370	18752
pmp001270	LysoPC 16:1	Lipids	26168	16550	20277	21420	16326	17954
pmn001681	1-(4-Methoxyphenyl)-1-propanol	Phenolic acids	14237	15309	31970	17033	18434	14170
Lmhn002926	p-Coumaroylmalic acid	Phenolic acids	29106	19604	26280	16479	12196	15084
Lmhp009590	LysoPC 17:1	Lipids	25717	16241	18691	13014	16794	20315
pmp001312	6-Hydroxykaempferol-3,7,6-O-triglycoside	Flavonoids	21961	24750	18043	15619	17587	16785
pme0008	L-Citrulline	Amino acids and derivatives	18899	15432	18539	14756	13854	16394
YC512119	Phytosphingosine	Others	14545	14667	15533	21980	17597	17017
mws0242	SubericAcid	Organic acids	20946	19040	18417	15785	13494	16699
mws1421	α-Viniferin	Others	9	9	9	6664.2	7078.4	5820.9
pme0181	1-Methylhistidine	Amino acids and derivatives	18337	16600	15395	12168	17153	21389
Lmhn003074	Feruloylmalic acid	Phenolic acids	24084	16439	20428	5334.5	4793.6	4127.5
pme1730	D-Erythronolactone	Organic acids	17376	15946	18541	15345	15716	15953
mws1383	Lumichrome	Alkaloids	10797	14727	12622	12377	13908	13436
mws2523	Trehalose 6-phosphate	Others	14228	14729	15487	16558	15835	17611
mws0852	Methotrexate	Nucleotides and derivatives	9648.3	8553.5	12167	15521	12766	16267
pmb2260	LysoPC 15:1	Lipids	10832	9559	11509	14014	14538	15792
pme0033	Hypoxanthine	Nucleotides and derivatives	18130	13841	12261	11836	15296	14162

mws0124	N-(3-Indolylacetyl)-L-alanine	Amino acids and derivatives	46473	39102	55441	5421.1	4885.3	5737.1
pmb2804	13-HPODE	Lipids	10840	14737	11654	12433	12650	11483
mws0361	Palmitoleic Acid	Lipids	10357	10365	10530	10523	12729	13615
mws0572	5-Methylcytosine	Nucleotides and derivatives	4114.5	6284	9038.2	20872	16681	17480
pme0120	5-Aminovaleric acid*	Amino acids and derivatives	6798.2	4184.1	7445.4	5519.4	14422	14701
pme1738	3-Carbamyl-1-methylpyridinium(1-Methylnicotinamide)	Others	5588.8	10414	8712.3	11380	12302	14962
pmb2657	Argininosuccinic acid	Organic acids	10840	12845	12465	10619	11664	13655
mws0981	Isoxanthopterin	Nucleotides and derivatives	10227	9918.9	13255	12486	19148	11157
pmd0160	LysoPE 16:0(2n isomer)	Lipids	13319	4445.2	4734.9	7939.6	7781.4	11169
pme1187	5-Methyluridine	Nucleotides and derivatives	13708	13577	14534	9542.3	10760	13407
mws1589	Panose*	Others	20056	15603	11751	11404	7027.2	10252
Hmlp001371	Cyclo(Tyr-Ala)	Amino acids and derivatives	7813	10626	11001	12291	8968.8	10196
pmb2857	L-Glutamic acid O-glycoside	Amino acids and derivatives	3182.5	4494	9320.6	20731	16656	23051
mws5035	Leucylphenylalanine	Amino acids and derivatives	3463.3	7072.4	4606.1	10011	11155	12050
pmb3075	3-O-p-Coumaroylshikimic acid	Phenolic acids	13386	13100	12062	9234.9	12525	12947
Lmhp008718	LysoPC 17:2	Lipids	10035	7596.1	7682.9	8968.8	8600.8	10837

mws0057	Eriodictyol 7-O-glucoside	Flavonoids	7207.2	9973.9	6884.9	10185	9269.9	9000.5
pmb2799	12,13-EODE	Lipids	8023.9	14745	9105.6	7624.8	8331.3	8174.5
Hmjn008136	Camaldulenic acid	Terpenoids	9	9	9	53249	41390	29184
pmb0962	Lysine butyrate	Amino acids and derivatives	3901.3	5135.8	2445.3	5455.3	4082.9	5795
mws1078	Anthranilic Acid	Phenolic acids	9550.8	10215	10334	9661.1	7225.7	8498.6
pmb1096	Indole	Alkaloids	3654.8	5519.5	5667.9	7477.9	4614.4	5737
mws2218	Caffeine	Alkaloids	9	24162	9	9	9	16446
mws1420	ε-Viniferin	Others	6457.4	20230	11591	9	9	9
pmd0136	LysoPC 18:0(2n isomer)	Lipids	8542	3963.5	4217	7133.6	5467.9	9018.9
pme2237	Dulcitol*	Others	8191	9700	9148.7	8246.2	8549.6	9073.8
pmf0297	1-Eicosanol	Lipids	6342.4	9201.6	7011	7405.5	8370.2	7264.2
Hmbn002174	4-Hydroxyacetophenone	Phenolic acids	8551.7	7831.2	9441.1	5587.2	8987.9	4535.9
Hmcp002268	Limocitrin 3-rhamnoside	Flavonoids	5033.9	4381.7	4773.5	3056	2516.6	9334
mws0596	3-Hydroxyanthranilic acid	Organic acids	9	9	9	8505.4	6788	6743.4
pmb2791	9-HpOTrE	Lipids	7924.4	8360.1	7316.6	7233.6	6888.5	7508
mws1212	Methyl ferulate	Phenolic acids	11467	6222.7	8351.5	2517.2	3155.8	2428.5
pmb2640	Lauric acid	Lipids	5475.4	6895.4	7096.5	6016.2	6166.1	5561.2
mws0289	LysoPE 18:1	Lipids	6826.2	9	9	4431.7	4571.3	7546.2
pme0500	D-(+)-Melezitose*	Others	7501.7	6749.2	4845.4	5735.2	2372.8	4855.5
pmn001691	9,12,13-Trihyroxy-10,15-octadecadienoic acid	Lipids	8780.4	6231.5	8354	4184.4	4989.7	4763.5
pmn001695	Trihydroxycinnamoylquinic acid	Phenolic acids	8102.7	10390	15394	9	9	6352.8
mws0120	Choline alfoscerate	Lipids	5016.3	3451.5	4055.3	6208.6	9889.8	14149
Lmhp008801	LysoPE 18:3	Lipids	5422.2	2457.3	2584.8	3955.2	4663.2	7149.9

pme2890	L-Homocystine	Amino acids and derivatives	4722.7	4000.6	4706.4	6525.7	3953.9	5857.1
Lmhp009129	LysoPC 15:0	Lipids	4816.3	3020.8	3658.3	3993.8	2288.4	3963.1
mws0636	Phe-Phe	Amino acids and derivatives	4516.8	4130.4	4353	5636	4925.7	6070.8
pmb2786	9-HOTrE	Lipids	3706	3880.2	3453.1	2450	3221.2	6360.3
pmb1283	L-Glutaminyl-L-valyl-L-valyl-L-cysteine	Amino acids and derivatives	3585.7	4148.8	5144.4	9	9	9

(treatments: R12 and R12E)

Index	Compounds	Class I	R12_1	R12_2	R12_3	R12E_1	R12E_2	R12E_3
mws0470	Methylmalonic acid*	Organic acids	45460000	50969000	51748000	41359000	43552000	42781000
mws0192	Succinic acid*	Organic acids	41685000	46044000	48213000	40285000	41762000	39935000
pme0230	Adenosine	Nucleotides and derivatives	20094000	20743000	20691000	22596000	21269000	22212000
pme0021	L-Phenylalanine	Amino acids and derivatives	19753000	19192000	19041000	18555000	18324000	19083000
mws0277	Kinic acid	Organic acids	19108000	18273000	17775000	17693000	17984000	16339000
pmb0789	Pyridoxine-5'-O-glucoside	Others	19335000	20846000	20248000	18457000	20580000	17058000
mws0216	Trans-4-Hydroxy-L-proline	Amino acids and derivatives	17508000	18316000	14511000	7701500	10082000	15159000
mws0281	Citric Acid	Organic acids	17387000	16237000	15815000	13830000	12801000	13310000
pmc0274	6-Methylmercaptopurine	Nucleotides and derivatives	14188000	14290000	14316000	13917000	13139000	14423000
Rfmb324	Trigonelline	Alkaloids	9717500	9823600	9596500	10769000	11998000	11073000
pme0006	L-Proline	Amino acids and derivatives	11870000	11757000	11658000	11000000	11775000	12236000

pmn001578	Hexadecanoic acid	Lipids	9018000	7600900	8753300	6471600	7631500	6932700
mws0119	Myristic Acid	Lipids	7649200	7032800	7362500	6653500	6512300	6248800
pmp001287	N-Benzylmethylene isomethylamine	Alkaloids	6718300	6779600	6786200	6559000	6409100	7240800
pme1474	5'-Deoxy-5'-(methylthio)adenosine	Nucleotides and derivatives	7468100	7316200	7137100	6653500	7311800	7565000
mws1489	Stearic Acid	Lipids	7357200	5793600	8139700	6801500	6981600	5731900
mws0256	L-Valine*	Amino acids and derivatives	5794100	5618500	5733600	5589400	6353200	5714600
Zmhn001970	Piceid	Others	7866600	8918600	8079800	7973200	5288700	10352000
pmb0855	LysoPC 16:0	Lipids	7626900	7647900	7798300	6731400	4771600	9206500
mws0250	L-(-)-Tyrosine	Amino acids and derivatives	4893200	4838400	5322300	5434200	5292300	5757300
pme1383	Pyridoxine	Others	6514600	7332300	7300900	5585000	6651000	4591400
mws0366	γ-Linolenic Acid	Lipids	4117200	4111800	4277800	3339000	4262800	3867100
pmp001198	6-Deoxyfagomine	Alkaloids	4205400	4325300	4766800	4479600	4531000	5051600
pmb3101	2-Isopropylmalate	Organic acids	2682200	2726100	2792000	4113100	6118200	3857800
Hmsn000210	Methyl 7,10-hexadecadienoate	Lipids	3471000	3568200	3728500	3862500	3761300	3331400
pme2380	A-Ketoglutaric acid	Organic acids	3235000	3027700	2927200	3315000	3397800	3262100
pma3101	Nicotinate D-ribonucleoside	Others	3571000	3525100	3629900	3350600	3211300	3443200
mws1337	D-Pantothenic Acid	Others	3522300	3493000	3530100	3491500	3776600	3456700
pmp001273	LysoPC 18:2	Lipids	4513200	4562600	4523600	4055700	2704800	5333100
pmb2922	Uridine 5'-diphospho-D-glucose	Nucleotides and derivatives	3179700	3374300	3181800	3391000	3736300	3290700
pme2914	3-Hydroxy-3-methylpentane-1,5-dioic acid	Amino acids and derivatives	2333200	2589400	2469800	3126100	3174800	3670200

pme3011	γ-Aminobutyric acid*	Organic acids	3106000	2873500	3006900	3084700	3441100	3326100	
mws0282	L-Tryptophan	Amino acids and derivatives	3553000	3078900	3399200	3052100	2784600	3407600	
Zmhn001414	Resveratrol-O-diglucoside	Others	3554200	3354300	3213200	3288900	2292200	3150900	
pmn001419	1-O-[(E)-p-Cumaroyl]-β-D-glucopyranose	Phenolic acids	3108700	3231000	3193600	2981800	2286900	3169700	
pme0040	Adenine	Nucleotides and derivatives	2216900	2285800	2282100	2543400	2279300	2453100	
pmb1650	Octadeca-11E,13E,15Z-trienoic acid	Lipids	2757100	2204300	2058300	2283600	2381300	2337100	
pmn001420	1-O-[(E)-Caffeoyl]-β-D-glucopyranose	Phenolic acids	2197500	2232500	2274700	2393700	2037100	3186500	
pme3017	2-Aminoisobutyric acid*	Amino acids and derivatives	2425400	2637600	2402600	2533700	2306400	2714400	
pme0014	L-Glutamic acid	Amino acids and derivatives	2210400	1860400	1905300	1859000	2387800	1872400	
Rfmb319	Pipecolic acid	Amino acids and derivatives	2285700	2325400	2180000	2481600	2298400	2746100	
pme0193	L-Glutamine	Amino acids and derivatives	1802500	1828800	1792400	1902700	1749400	2067000	
mws5040	Turanose	Others	2533500	3338100	2357100	1812700	1977000	2366200	
pme1178	Guanosine	Nucleotides and derivatives	2220700	2071400	1953000	2075800	1978500	1915800	
pmb3081	Glucarate O-Phosphoric acid	Others	2519900	2545800	2117500	2065300	1536900	2112900	
pme0026	L-(+)-Lysine	Amino acids and derivatives	1824900	1774100	1721000	1728800	1800800	1984800	

pme3184	2'-Deoxyadenosine-5'-monophosphate	Nucleotides and derivatives	1979300	2398700	2421400	2239300	2242500	1903200
mws0260	L-(+)-Arginine	Amino acids and derivatives	2139200	1836000	2001400	1979100	1782200	2307200
pme3033	N,N-Dimethylglycine	Amino acids and derivatives	1708000	1644600	1627700	1774700	1765100	1931500
pme3096	Aminomalonic acid	Organic acids	1778800	1952500	2002300	1559400	1717600	1550400
mws0219	L-AsparticAcid	Amino acids and derivatives	1408200	1372000	1379200	1480100	1615000	1498900
pmp001086	5-Hydroxymethylfurfural	Phenolic acids	1297000	1282200	1188400	1316700	1398100	1334000
mws0275	L-(-)-Malic acid	Organic acids	1202700	1323600	1288100	1334000	1282800	1369200
Lmhp010190	LysoPC 18:1(2n isomer)	Lipids	1996400	2032200	1986800	1474600	995080	2114200
mws0866	D-Glucose 6-phosphate	Others	1306400	1359900	1299400	1361600	1478000	1254600
mws0628	4-Hydroxybenzaldehyde	Phenolic acids	1197000	1288500	1275400	1138700	1045100	1173800
pme0534	Gluconic acid	Others	947620	1028500	1047200	816040	771930	827000
mws0983	N-Oleoylethanolamine	Others	879640	741870	811100	780510	835880	568060
pme3163	D-Sedoheptuiose 7-phosphate	Others	1007900	1115500	1088500	1175200	926690	962640
pmc0066	2'-Deoxyinosine-5'-monophosphate	Nucleotides and derivatives	832330	842940	816290	846280	934670	970020
mws0227	L-Leucine*	Amino acids and derivatives	894500	968650	928520	887760	863180	1015700
pmn001367	Protocatechuic acid-4-glucoside	Phenolic acids	977530	1062500	968400	822620	788740	948390
pme1210	L-Methionine	Amino acids and derivatives	827900	819200	865440	1003700	913960	963110
mws0671	L-Homoserine*	Organic acids	927940	941550	936390	825120	784690	883290

mws0567	4-Guanidinobutyric acid	Organic acids	778530	741510	721240	849150	924340	941330
pme0516	Inositol	Others	867010	809740	814370	969710	878920	757930
pme1436	p-Coumaric acid	Phenolic acids	505390	484790	618520	411250	470910	638670
Zmxn001997	Isosalicylic acid O-glycoside	Phenolic acids	705170	752030	737040	638410	542600	847220
pme2693	N-Acetylputrescine	Alkaloids	602270	653960	635010	815790	649700	805690
pmb2826	Citramalate	Organic acids	601340	734720	818140	714570	793060	693210
pme3337	N6-Succinyl Adenosine	Nucleotides and derivatives	837080	801070	801750	777110	780520	796170
pmb0501	Agmatine	Alkaloids	616380	591880	615970	784490	664100	821050
pmn001517	3,4,5-Trimethoxyphenyl- β -D-Glucopyranoside	Phenolic acids	740780	704400	744960	613710	681960	768630
pme2266	Biotin	Others	954520	956890	958000	769370	869540	670320
pme3705	D-Glucoronic acid	Others	570060	544290	593860	436800	479600	473750
pmb0981	Adenosine 5'-monophosphate	Nucleotides and derivatives	612390	651090	700130	756690	882640	652560
mws0237	Anchoic Acid	Organic acids	736170	720050	725170	663920	743350	654070
Lmtn002565	1'-O-Vanilloyl- β -D-glucoside	Phenolic acids	836260	822910	816930	1257500	1233200	1315500
HmLn000873	2-O-Galloyl- β -D-glucose	Tannins	114180	127980	137960	1377400	1376200	2276600
pmp001281	LysoPC 18:1	Lipids	943360	947940	966340	758180	503190	1161800
Lmtn002233	Androsin	Others	619600	567560	591190	465460	344350	577190
pme3732	Cytidine	Nucleotides and derivatives	554500	492780	551500	515970	491670	534440
mws0232	Riboflavin	Others	453690	435010	472870	569740	576060	441860
pme0281	Terephthalic acid	Phenolic acids	465920	454480	481400	532700	466360	571650
pmb0865	LysoPC 18:3(2n isomer)	Lipids	634060	650160	622610	515350	364690	663950

pme0274	6-Aminocaproic acid	Organic acids	593300	511250	491780	399760	493130	521970
pmb0764	4-Methyl-5-thiazoleethanol	Others	561520	634470	609820	398880	558760	355910
mws0752	Undecylic Acid	Lipids	548840	477780	486470	433610	446950	450210
mws0749	4-Hydroxybenzoic acid	Phenolic acids	468980	461800	468820	389100	379850	399020
mws5038	Isomaltulose*	Others	581420	530690	548350	424260	426370	408550
pme0519	D-(+)-Sucrose*	Others	590060	473060	482150	501960	462720	339610
pmn001706	2-Hydroxyoleanolic acid	Terpenoids	83930	92104	86024	89725	89304	80983
pme2746	Flavin adenine dinucleotide(FAD)	Nucleotides and derivatives	445640	476630	477210	450810	459620	409400
pmp001285	Phthalic anhydride	Phenolic acids	471520	371290	339930	414170	432230	389220
pme1109	Guanine	Nucleotides and derivatives	418150	399990	468200	413070	359360	388790
mws0376	Fumaric acid	Organic acids	420850	439660	401390	385480	376860	404430
mws4170	D-Glucose	Others	494670	478800	486210	321980	321160	340410
pme3313	D-Fructose 6-phosphate	Others	522170	506640	449450	387800	384810	329780
mws1050	O-Acetylserine	Amino acids and derivatives	374920	373450	380770	265410	361870	286470
pme1975	Malonic acid	Organic acids	346600	267450	213130	313290	300960	348330
pmb0874	LysoPE 18:2(2n isomer)	Lipids	652990	657530	651840	410750	226830	926820
mws1038	(R)-Pantetheine	Others	361910	450180	368370	366900	323590	297090
mws0230	L-(-)-Threonine*	Amino acids and derivatives	343550	378460	365240	339060	341580	351790
pmb0530	Nicotinic acid adenine dinucleotide	Nucleotides and derivatives	400360	283380	350230	352390	348820	383400

mws4134	Oxidized Glutathione	Amino acids and derivatives	309740	295050	279130	345300	375220	288520
mws2623	11-Octadecanoic acid(Vaccenic acid)	Lipids	298380	244740	258230	187340	283060	178890
mws0254	L-Histidine	Amino acids and derivatives	384940	349450	326940	307580	293020	311680
pmb3894	Di-O-methylquercetin	Flavonoids	303950	313790	304300	252270	201170	277230
Hmtp000776	4,5,6-Trihydroxy-2-cyclohexen-1-ylideneacetonitrile	Alkaloids	273980	267210	269600	270420	245330	270890
pmb0854	LysoPC 18:3	Lipids	300160	322060	323120	278920	196810	357230
pme2598	3,4-Dihydroxybenzeneacetic acid	Phenolic acids	329760	331150	323590	228570	194050	227040
mws0473	2-Methylsuccinic acid	Organic acids	219620	207300	198240	195480	226320	199210
pmn001694	9,10,13-Trihyroxy-11-octadecadienoic acid	Lipids	267960	273810	273210	218930	175550	242200
mws0133	Nicotinamide	Others	353680	340900	331760	165970	230510	197420
pmb0786	Glucosamine	Others	246800	230980	238210	226190	232100	211730
pmd0132	LysoPC 16:0(2n isomer)	Lipids	306070	308430	314200	326810	233110	441920
pmn001495	Hexadecanoic acid 2,3-dihydroxypropyl ester	Lipids	259310	263900	269450	214680	169890	238020
mws0248	Uridine	Nucleotides and derivatives	209790	203860	212750	199800	165930	188740
Rfmb320	1,2-N-Methylpipecolic acid	Amino acids and derivatives	195760	232940	201720	199660	231350	246460
pme0122	N6-Acetyl-L-lysine	Amino acids and derivatives	141670	149800	163060	167100	181750	202690
mws0258	L-Isoleucine*	Amino acids and derivatives	167290	177650	165110	127880	167440	153960

mws0668	Xanthosine	Nucleotides and derivatives	338010	328250	375360	168950	165180	167410
mws0147	3-Hydroxy-3-methyl butyric acid	Organic acids	205700	236400	216890	179300	179550	176240
Hmtn001302	Glucosyloxybenzoic acid	Phenolic acids	282410	277880	282430	144470	181030	200010
mws1333	Melibiose*	Others	224720	216090	172580	213170	175500	162530
Zmhn002422	Feruloyl glucose	Phenolic acids	181730	191190	171840	179850	138770	234400
Zmhn001926	Salicylic acid O-glycoside	Phenolic acids	265980	267720	297500	158920	171320	180210
pmb2778	9,10-EODE*	Lipids	204190	198420	204870	168920	237660	293020
mws0736	N-Glycyl-L-leucine*	Amino acids and derivatives	169470	175200	171420	200520	165720	195080
pme0490	Nicotinic acid	Others	177620	187990	182370	161930	178450	198580
mws0208	Adipic Acid	Organic acids	217630	216480	220760	186630	197130	189420
pme1014	Menaquinone (K2)	Others	220570	234040	179370	170590	197080	234200
pmb0964	Isopentenyladenine-7-N-glucoside	Nucleotides and derivatives	208110	147340	186700	182520	202940	162860
pmn001511	3-Hydroxy-5-Methylphenol-1-Oxy-β-D-Glucose	Phenolic acids	232530	233370	238320	254410	232660	274170
pmb0449	2-Aminoadipic acid (L-Homoglutamic acid)	Amino acids and derivatives	157110	181340	161900	114360	144680	160010
mws5041	Glycylisoleucine*	Amino acids and derivatives	146020	154730	144480	154620	133750	176550
pmb3099	Diethyl phosphate	Organic acids	135370	31515	3128.3	129410	114900	141080
pme3083	2-(Formylamino)benzoic acid	Phenolic acids	114450	143920	118960	104290	105430	143840
pme3967	2-(Dimethylamino)guanosine	Nucleotides and derivatives	113180	109160	118840	141960	142550	132600

mws0609	Guanosine 3',5'-cyclic monophosphate	Nucleotides and derivatives	186380	185990	197090	160890	172490	155250
pmb1912	10-Formyl-THF	Alkaloids	134110	116250	137790	123430	125640	146540
mws0458	Vanillin	Phenolic acids	134100	107770	101990	111050	103340	84600
pme0010	L-Serine	Amino acids and derivatives	125710	157590	158670	128290	111140	132160
pme0256	Xanthine	Nucleotides and derivatives	132470	101580	107830	100030	92407	94875
pmb2497	4-Hydroxy-3-methoxymandelate	Phenolic acids	196460	155080	134860	127160	124330	109710
pme1216	2-Picolinic acid	Organic acids	137960	116970	148850	98303	109150	86296
pme0253	N-Acetyl-L-leucine	Amino acids and derivatives	68561	71854	76937	52835	49161	50036
pmb0464	Aspartic acid di-O-glucoside	Amino acids and derivatives	171250	143050	127810	121630	131460	126360
pmb0876	LysoPE 16:0	Lipids	233680	240360	234790	138750	64189	437000
pme3961	Deoxyadenosine	Nucleotides and derivatives	129180	118510	115270	114490	115200	105190
pmb3107	Syringic acid O-glucoside	Phenolic acids	51986	43963	49475	105590	120380	137630
Smsn001839	Dihydrocornin	Terpenoids	135220	149240	141230	117970	125690	138860
mws0126	LysoPC 18:0	Lipids	186960	150800	149340	161580	82654	279470
pme2735	S-Adenosylmethionine	Amino acids and derivatives	100630	89725	98848	113730	108650	88018
mws0675	β-Nicotinamide mononucleotide	Nucleotides and derivatives	73671	66881	87755	102940	150440	87442
pme0183	2-Hydroxy-6-aminopurine	Nucleotides and derivatives	110890	109580	96964	89574	79344	83691

pme2755	N-Acetyl-D-glucosamine	Others	116400	105490	107370	98681	108470	105750
pme2651	NADP (Nicotinamide adenine dinucleotide phosphate)	Nucleotides and derivatives	126860	103530	85127	96662	89987	99494
mws1080	Galactinol*	Others	142840	141730	154150	123520	95924	124610
pmp001276	2,3-Dihydroxypropyl-9,12,15-octadecatrienoate-hexose-hexose	Lipids	118020	109040	101930	67350	70924	68377
pmf0440	4-MethoxycinnaMaldehyde	Phenolic acids	117920	41859	26260	102110	81009	110880
mws5042	Glycylphenylalanine	Amino acids and derivatives	102160	96877	99878	93178	82427	91688
mws0255	Cytosine	Nucleotides and derivatives	91868	85052	83696	86467	94480	99202
mws1060	9-(β-D-Arabinofuranosyl)hypoxanthine	Nucleotides and derivatives	175360	179520	165610	55576	62139	63862
pmn001690	3-Hydroxy-4-isopropylbenzylalcohol 3-glucoside	Phenolic acids	118940	106150	117310	111660	132330	125110
pme2559	N-Acetylaspartate	Amino acids and derivatives	64535	67774	63213	83141	84919	82320
mws2212	Caffeic acid	Phenolic acids	89149	87091	83093	70229	70821	80744
pme0001	Hesperetin 7-O-neohesperidoside(Neohesperidin)	Flavonoids	4790.1	5137.6	3770	9	9	9
mws0179	Chlorogenic acid methyl ester	Phenolic acids	79330	123120	119880	73719	107100	96623
Lmhp011562	1-α-Linolenoyl-glycerol*	Lipids	58914	58064	57314	54248	65292	68795
mws0884	Cyclic AMP	Nucleotides and derivatives	78560	76885	75173	83309	74557	77850
pmb0889	Punicic acid	Lipids	81308	78040	79421	70814	94286	125690

Rfmb090	13-Hydroxy-9,11-octadecadienoic acid*	Lipids	85895	78506	84720	64026	99508	124250
mws1491	Linoleic acid	Lipids	67198	65184	64073	91658	86711	73538
pmb2561	N-Acetylmethionine	Amino acids and derivatives	77873	75922	95268	38949	94980	34435
mws0972	5-Hydroxyhexanoic acid	Organic acids	80352	78583	85026	125680	100330	71137
pme0075	N-Acetyl-L-glutamic acid	Amino acids and derivatives	76150	61246	74131	57889	64495	58118
Lmhp012042	2-Linoleoylglycerol*	Lipids	59799	53150	54478	47770	54349	65996
pme1419	L-Methionine methyl ester	Amino acids and derivatives	65707	62601	67359	68364	74631	67067
pmn001688	9S-Hyroxy-10E,12E-octadecadienoic acid*	Lipids	75094	73129	74758	58506	91943	112310
mws0359	Pentadecanoic Acid	Lipids	85556	87166	87374	70195	70208	74571
Lmhp011388	2- γ -Linolenoyl-glycerol*	Lipids	52033	52777	45673	39705	44853	54854
pma0149	Sinapoyl malate	Phenolic acids	47764	50899	56369	55760	49003	56851
mws0520	N-Acetyl-L-tyrosine	Amino acids and derivatives	16847	19081	18775	16626	16805	14163
pmb0374	Aminopurine	Alkaloids	54786	61017	50996	70795	65510	56084
mws0191	Betaine	Alkaloids	51296	51467	53333	62785	51674	60944
pme3186	DL-Glyceraldehyde 3-phosphate	Organic acids	67285	75833	92200	72380	63836	45177
pme0295	4-Acetamidobutyric acid	Organic acids	55870	66680	63213	53145	59517	61831
mws5037	Alanylleucine	Amino acids and derivatives	40843	44068	37050	45184	45589	40812
pme1002	L-Tyramine	Amino acids and derivatives	53683	48182	55458	9477.8	26308	6763.9

Lmhp112042	1-Linoleoylglycerol*	Lipids	52238	46516	43008	42368	53206	51486
pme2117	Adenosine 5'-Diphosphate	Nucleotides and derivatives	57461	75710	63961	41462	50163	44198
mws0847	1-Methyladenine	Nucleotides and derivatives	55193	48375	42294	55163	56734	50959
mws0146	Nicotinic Acid Methyl Ester(Methyl Nicotinate)	Alkaloids	46923	51056	48883	60958	66045	64501
mws0001	L-Asparagine	Amino acids and derivatives	71548	75429	61702	45798	51747	58095
mws1200	Trans-4-Hydroxycinnamic Acid Methyl Ester	Phenolic acids	28021	29954	30006	35945	37067	52735
mws0341	(S)-(-)-2-Hydroxyisocaproic acid	Organic acids	62147	55818	55044	41826	38541	39860
pme3388	H-HomoArg-OH	Amino acids and derivatives	55067	56452	56810	53909	42247	67753
pme1286	S-(5'-Adenosy)-L-homocysteine	Amino acids and derivatives	49472	59658	46529	48226	47141	53250
pme2529	1,5-Anhydro-D-glucitol	Others	51337	42946	43844	55936	69453	49393
pmf0359	Skimmin	Lignans and Coumarins	50737	48191	44397	45693	38486	53062
pmb0881	LysoPE 18:2	Lipids	80674	86726	95501	48892	27686	167590
pme0170	N-α-Acetyl-L-arginine	Amino acids and derivatives	29659	31074	31006	28655	24657	30483
pme0278	2,6-Diaminooimelic acid	Amino acids and derivatives	33599	33076	38931	35885	46300	49750
pmb2406	LysoPC 17:0	Lipids	64418	63009	65916	58062	31903	86745
Lmtn002796	Aromadendrin 7-glucoside	Others	49991	45446	52335	34063	42587	72545

pme2596	4-Pyridoxic acid	Others	40490	43392	44464	37995	36937	36092
mws0629	Asp-phe	Amino acids and derivatives	43501	43694	42577	46226	42411	43064
pmn001606	Eicosenoic acid	Lipids	38025	37733	40627	30055	43096	29752
pme3382	N-Acetylthreonine	Amino acids and derivatives	42647	33254	34066	32594	34407	30785
Lmyn000239	Cordycepic acid*	Others	42364	38626	48737	40199	28997	40790
mws0193	L-Homocitrulline	Amino acids and derivatives	36390	34729	30479	23943	24868	33323
pme1184	Deoxyguanosine	Nucleotides and derivatives	42703	43383	44139	42678	45561	42589
pmp001275	3-Hydroxypropyl palmitate glc-glucosamine	Alkaloids	32027	35125	32374	29900	26873	28285
pmn001668	Apigenin-3-O-α-L-rhamnoside	Flavonoids	43608	44780	50659	36847	39929	41183
Zmhn002227	Sinapic acid-glycoside	Phenolic acids	46037	49338	42137	25809	32847	43784
mws4176	Alanylphenylalanine	Amino acids and derivatives	27879	30315	24479	35377	30038	40929
pmp001282	Propyl2-(trimethylammonio)ethyl phosphate	Others	58525	54028	57505	29821	20372	46539
pmb0197	N2-methylguanosine	Nucleotides and derivatives	23254	26911	33832	27282	34198	24225
Lmdp003090	Dihydroquercetin(Taxifolin)	Flavonoids	37835	33109	34712	44669	42464	51686
Lmhn002683	p-Coumaroylcaffeoyltartaric acid	Phenolic acids	12338	16733	11692	10660	11168	10934
pme1194	Deoxycytidine	Nucleotides and derivatives	23526	27919	28551	37921	35502	28275

pme3174	Cytidine 5'-monophosphate(Cytidylic acid)	Nucleotides and derivatives	24103	39806	39465	35525	36486	29008
YC512118	Octadecenoic amide	Others	25961	137510	109990	15504	5851.4	48245
pme0264	Thymidine	Nucleotides and derivatives	25652	25572	23697	33566	26804	31978
mws1499	D-(-)-Arabinose	Others	27495	32483	22193	19192	17926	22246
pmb2792	13-HOTrE(r)	Lipids	67299	41726	71425	29361	32326	22945
pmb0856	LysoPE 18:1(2n isomer)	Lipids	49851	48893	46077	26370	18800	66080
pmb2363	MAG(18:1)	Lipids	33666	32118	30846	18690	13242	17192
pme1021	D-(+)-Glucono-1,5-lactone	Others	20732	26381	27149	40809	27693	28987
pmb2653	D(+)-Melezitose O-rhamnoside	Others	43417	37522	43283	23264	22489	19394
pme3968	7-Methylguanine	Nucleotides and derivatives	23580	30102	26719	24106	26666	21343
Lmhp008589	LysoPE 18:3(2n isomer)	Lipids	43174	42146	44270	28078	16284	59781
mws0177	2-Furanoic acid	Organic acids	25291	30040	23693	14670	20513	12456
pmn001336	Aloeemodin-8-O-D-glucopyranoside	Quinones	28418	28769	27364	23848	22257	24649
pme0195	L-Cysteine	Amino acids and derivatives	27667	20274	33708	22172	26153	25425
pmn001352	6-Hydroxyrumicin-8-O-D-glucopyranoside	Quinones	16456	17960	15096	21747	22612	38044
Lmdp003994	Wistin (6,4'-Dimethoxyisoflavone-7-glucoside)	Flavonoids	33731	32821	34964	20852	21147	21770
pmb2654	Anthranilate O-hexosyl-O-hexoside	Phenolic acids	30065	26934	28988	22319	20986	17914
pme0241	Benzoic acid	Phenolic acids	19804	23809	21699	20620	18210	16373
mws0011	Syringin	Phenolic acids	33866	31299	17815	16375	14444	50253

Lmtn003598	3-Prenyl-4-O-β-D-glucopyranosyloxy-4-hydroxyl-benzoic acid	Phenolic acids	11738	17748	20104	15522	14120	15450
pmb0801	4-Pyridoxic acid O-hexoside	Others	20311	20260	18852	17273	20597	24291
Hmpp003242	Isorhamnetin-3-O-β-D-glucoside	Flavonoids	29627	37717	41792	17144	15619	21358
pme2527	L-Ornithine	Amino acids and derivatives	17637	19453	19159	21547	19697	23354
pmp001270	LysoPC 16:1	Lipids	29000	29363	30676	20090	18541	28233
pmn001681	1-(4-Methoxyphenyl)-1-propanol	Phenolic acids	17406	21486	17917	19020	19777	16993
Lmhn002926	p-Coumaroylmalic acid	Phenolic acids	16310	11556	13681	13242	13581	24766
Lmhp009590	LysoPC 17:1	Lipids	26790	23393	26220	18372	9233.9	23505
pmp001312	6-Hydroxykaempferol-3,7,6-O-triglycoside	Flavonoids	14599	14826	12700	19130	23702	26940
pme0008	L-Citrulline	Amino acids and derivatives	18256	16466	19311	16656	11343	17986
YC512119	Phytosphingosine	Others	16955	21112	16862	17888	15897	21346
mws0242	SubericAcid	Organic acids	13081	14439	17865	20011	17498	16959
mws1421	α-Viniferin	Others	31388	90603	131660	9	9	9
pme0181	1-Methylhistidine	Amino acids and derivatives	10929	11882	15382	16053	18601	21293
Lmhn003074	Feruloylmalic acid	Phenolic acids	18429	14725	16013	8966	10416	17981
pme1730	D-Erythronolactone	Organic acids	17194	15592	15970	14860	18805	16293
mws1383	Lumichrome	Alkaloids	18141	18192	18790	14802	14719	17314
mws2523	Trehalose 6-phosphate	Others	12178	16448	16277	14080	15110	16565

mws0852	Methotrexate	Nucleotides and derivatives	15666	17575	16633	14207	18552	14808
pmb2260	LysoPC 15:1	Lipids	12618	12612	13634	18527	16934	15806
pme0033	Hypoxanthine	Nucleotides and derivatives	12275	11633	13733	13961	12243	13506
mws0124	N-(3-Indolylacetyl)-L-alanine	Amino acids and derivatives	8285.8	12262	14717	7706.8	7890.8	6835.5
pmb2804	13-HPODE	Lipids	12741	13436	13416	10775	16599	15044
mws0361	Palmitoleic Acid	Lipids	14252	17038	13279	11653	13426	13945
mws0572	5-Methylcytosine	Nucleotides and derivatives	9786.2	10462	11286	11206	11375	14242
pme0120	5-Aminovaleric acid*	Amino acids and derivatives	9459.3	14773	8686.7	14743	14934	15235
pme1738	3-Carbamyl-1-methylpyridinium(1-Methylnicotinamide)	Others	15767	8165.4	9335.4	9166.8	10239	13251
pmb2657	Argininosuccinic acid	Organic acids	13825	11719	14989	11438	15774	10752
mws0981	Isoxanthopterin	Nucleotides and derivatives	10010	12490	11578	9322.6	8138.8	11801
pmd0160	LysoPE 16:0(2n isomer)	Lipids	20889	21591	20565	15359	7310.8	44641
pme1187	5-Methyluridine	Nucleotides and derivatives	11120	9289	10976	10364	12760	13282
mws1589	Panose*	Others	18341	16158	14415	10859	9671	10550
Hmlp001371	Cyclo(Tyr-Ala)	Amino acids and derivatives	18051	15043	14303	11947	6515.1	9844.8
pmb2857	L-Glutamic acid O-glycoside	Amino acids and derivatives	11987	14187	12917	9	6844.8	9

mws5035	Leucylphenylalanine	Amino acids and derivatives	5832.8	5249.2	4775.3	6961.9	7798.1	6271
pmb3075	3-O-p-Coumaroylshikimic acid	Phenolic acids	4676.4	5335.8	4135.1	6683.9	7108.6	10642
Lmhp008718	LysoPC 17:2	Lipids	10720	13126	12528	9485.1	7833.1	15309
mws0057	Eriodictyol 7-O-glucoside	Flavonoids	15756	17847	19075	9747.2	12556	15413
pmb2799	12,13-EODE	Lipids	8524.1	8039.7	7250.9	5074	8219.4	8650.5
Hmjn008136	Camaldulenic acid	Terpenoids	9	9	9	9	9	9
pmb0962	Lysine butyrate	Amino acids and derivatives	7666.5	5411.4	6396.4	8000.8	7538	7279.2
mws1078	Anthranilic Acid	Phenolic acids	11499	11262	10263	6775.1	9426.8	8157.8
pmb1096	Indole	Alkaloids	9257.5	10485	11799	8055.1	7095.1	12032
mws2218	Caffeine	Alkaloids	7970.3	7154.2	8226.1	12777	25201	9
mws1420	ε-Viniferin	Others	16686	36878	44758	9	9	9
pmd0136	LysoPC 18:0(2n isomer)	Lipids	13962	9561.6	10689	10968	6445.6	19533
pme2237	Dulcitol*	Others	4218.9	5690	7986.4	7438.3	7782.2	7394.4
pmf0297	1-Eicosanol	Lipids	8004.4	7682.7	5627.4	5221.1	13523	8900.7
Hmbn002174	4-Hydroxyacetophenone	Phenolic acids	8204.2	6556.6	4886.6	8092.8	7090.8	5892.9
Hmcp002268	Limocitrin 3-rhamnoside	Flavonoids	7163.8	7842.9	6789.4	3024.7	3081.1	3637.8
mws0596	3-Hydroxyanthranilic acid	Organic acids	7626.8	7659.4	11860	8694.4	9391.3	11953
pmb2791	9-HpOTrE	Lipids	6411	8373.3	6954.6	6622.1	6362.6	6065.1
mws1212	Methyl ferulate	Phenolic acids	5408.3	5565	5786.9	5368.6	6220.9	8849.6
pmb2640	Lauric acid	Lipids	7884.4	6418.2	5651.2	5064.8	5238.7	5813
mws0289	LysoPE 18:1	Lipids	11437	13277	13578	7055	9	22893
pme0500	D-(+)-Melezitose*	Others	7864.3	11133	10433	6856.7	4515.5	4737.3

pmn001691	9,12,13-Trihyroxy-10,15-octadecadienoic acid	Lipids	6505.6	6528.9	6188.7	5618.2	4695.7	6451.7
pmn001695	Trihydroxycinnamoylquinic acid	Phenolic acids	9	9	9	9	9	3928.3
mws0120	Choline alfoscerate	Lipids	6556.7	11604	10677	3510.5	5524	6873.8
Lmhp008801	LysoPE 18:3	Lipids	9784.8	8839.2	6743.6	6166.7	3735	13996
pme2890	L-Homocystine	Amino acids and derivatives	2417.6	3449.4	3031.1	4574.9	3286.6	2641.7
Lmhp009129	LysoPC 15:0	Lipids	6618.5	5928.2	5834	6658.6	5230.6	8153.2
mws0636	Phe-Phe	Amino acids and derivatives	3785.2	3169.5	3070.6	5011	4658.5	4674
pmb2786	9-HOTrE	Lipids	3509.5	3403.6	3409.8	2922.9	3296.2	5701.7
pmb1283	L-Glutaminyl-L-valyl-L-valyl-L-cysteine	Amino acids and derivatives	3066	2791.4	2863.7	3936.3	3700.1	4589.8
**(treatments: R32, R32E)**								
Index	Compounds	Class I	R32_1	R32_2	R32_3	R32E_1	R32E_2	R32E_3
mws0470	Methylmalonic acid*	Organic acids	36588000	33075000	35299000	36546000	37445000	41242000
mws0192	Succinic acid*	Organic acids	35359000	30623000	32783000	34266000	37650000	38025000
pme0230	Adenosine	Nucleotides and derivatives	24070000	22595000	23494000	23905000	22132000	20082000
pme0021	L-Phenylalanine	Amino acids and derivatives	22020000	20264000	21619000	20955000	21860000	20535000
mws0277	Kinic acid	Organic acids	17747000	16498000	17098000	21559000	19338000	19634000
pmb0789	Pyridoxine-5'-O-glucoside	Others	19193000	16366000	16299000	14982000	15490000	18619000
mws0216	Trans-4-Hydroxy-L-proline	Amino acids and derivatives	16345000	15539000	19212000	24762000	21149000	14786000

mws0281	Citric Acid	Organic acids	15733000	14396000	14280000	18553000	16342000	15846000
pmc0274	6-Methylmercaptopurine	Nucleotides and derivatives	16120000	14857000	15560000	16210000	16117000	15380000
Rfmb324	Trigonelline	Alkaloids	11754000	10332000	10994000	12596000	12376000	13249000
pme0006	L-Proline	Amino acids and derivatives	9131200	8286600	8916000	11574000	10975000	11890000
pmn001578	Hexadecanoic acid	Lipids	9855300	8126300	7713600	8110100	8583100	7969000
mws0119	Myristic Acid	Lipids	6733700	6398800	6173800	6965200	7682700	6564600
pmp001287	N-Benzylmethylene isomethylamine	Alkaloids	8103200	7517500	7800300	7519800	8096800	7592400
pme1474	5'-Deoxy-5'-(methylthio)adenosine	Nucleotides and derivatives	5899600	5340200	4754700	5572900	6155700	6095000
mws1489	Stearic Acid	Lipids	6973600	5048800	6291200	6869500	6845400	7258200
mws0256	L-Valine*	Amino acids and derivatives	8040400	6647000	7297500	9351700	8716900	8833700
Zmhn001970	Piceid	Others	11179000	10478000	9407800	4738800	5001800	4822200
pmb0855	LysoPC 16:0	Lipids	5831600	5471600	4037000	5754800	2538500	5490400
mws0250	L-(-)-Tyrosine	Amino acids and derivatives	8159000	5911200	6715600	6008200	6484900	6329000
pme1383	Pyridoxine	Others	5068200	4257600	4182600	5057300	5036700	6610100
mws0366	γ -Linolenic Acid	Lipids	5482200	4448500	4425000	4722800	6438400	4199900
pmp001198	6-Deoxyfagomine	Alkaloids	5449500	4851500	4954000	5457900	5136600	5755000
pmb3101	2-Isopropylmalate	Organic acids	7266800	5592500	5626200	5760300	5654800	6207600
Hmsn000210	Methyl 7,10-hexadecadienoate	Lipids	3661200	3977600	3874600	3849500	3327900	3236900
pme2380	A-Ketoglutaric acid	Organic acids	3469600	3385100	3410100	2699400	2877800	2616100
pma3101	Nicotinate D-ribonucleoside	Others	3372400	3812400	3299000	2963700	2772400	3423900

mws1337	D-Pantothenic Acid	Others	3581800	3280700	3390200	3441400	3162600	3442100	
pmp001273	LysoPC 18:2	Lipids	3574400	3213200	2323100	3247100	1473600	3266000	
pmb2922	Uridine 5'-diphospho-D-glucose	Nucleotides and derivatives	3397400	2749100	2979400	2383200	3313200	3152500	
pme2914	3-Hydroxy-3-methylpentane-1,5-dioic acid	Amino acids and derivatives	4036700	5024000	4433500	2911400	3127200	2611400	
pme3011	γ-Aminobutyric acid*	Organic acids	3239300	2668800	3038200	4155200	3223900	3310800	
mws0282	L-Tryptophan	Amino acids and derivatives	4154200	3026200	3222700	3523100	3366800	3549100	
Zmhn001414	Resveratrol-O-diglucoside	Others	1181600	1349700	1166400	2263000	2518800	2140500	
pmn001419	1-O-[(E)-p-Cumaroyl]-β-D-glucopyranose	Phenolic acids	2935500	5283200	3719400	2092400	3505400	2149500	
pme0040	Adenine	Nucleotides and derivatives	2542800	2601400	2655100	2297300	2579100	2065800	
pmb1650	Octadeca-11E,13E,15Z-trienoic acid	Lipids	3135300	3112100	2659400	1814000	2365200	2134500	
pmn001420	1-O-[(E)-Caffeoyl]-β-D-glucopyranose	Phenolic acids	2526200	3207600	3391300	2238500	2059900	2465700	
pme3017	2-Aminoisobutyric acid*	Amino acids and derivatives	2407000	2283000	2432700	2628600	2324300	2453800	
pme0014	L-Glutamic acid	Amino acids and derivatives	1827200	2021700	1964500	2729100	2465100	2308500	
Rfmb319	Pipecolic acid	Amino acids and derivatives	1806100	1877600	1814800	1340000	1639700	1727600	
pme0193	L-Glutamine	Amino acids and derivatives	2289400	1951200	2167100	2897300	2640600	2818600	
mws5040	Turanose	Others	1708100	1967900	2068800	1618600	1767400	1906600	

pme1178	Guanosine	Nucleotides and derivatives	2048800	1908200	2012100	2019100	1894400	1967200
pmb3081	Glucarate O-Phosphoric acid	Others	1551200	1469100	1768700	2225300	2212500	2217500
pme0026	L-(+)-Lysine	Amino acids and derivatives	2214500	1793700	2121600	2713500	2396500	2741900
pme3184	2'-Deoxyadenosine-5'-monophosphate	Nucleotides and derivatives	2103500	1870200	1703500	1572400	1698800	2125900
mws0260	L-(+)-Arginine	Amino acids and derivatives	1249000	1172900	1256200	1736700	2185200	3020200
pme3033	N,N-Dimethylglycine	Amino acids and derivatives	1735300	1591300	1707600	2498400	2013800	1927800
pme3096	Aminomalonic acid	Organic acids	1344100	1187300	1203800	1334000	1467200	1520700
mws0219	L-AsparticAcid	Amino acids and derivatives	1722800	1611600	1746900	1636200	1701600	1564000
pmp001086	5-Hydroxymethylfurfural	Phenolic acids	1454000	1683100	1283900	1188800	1183100	1457600
mws0275	L-(-)-Malic acid	Organic acids	1153800	1509400	1316000	1230800	1270600	1193600
Lmhp010190	LysoPC 18:1(2n isomer)	Lipids	1344700	1317700	857600	1335800	476250	1385400
mws0866	D-Glucose 6-phosphate	Others	1120900	1040100	963950	1168800	1201100	1243700
mws0628	4-Hydroxybenzaldehyde	Phenolic acids	941860	1059800	828320	1209600	1014800	1094600
pme0534	Gluconic acid	Others	830490	758440	753160	1089800	1017900	1030500
mws0983	N-Oleoylethanolamine	Others	1048000	1010800	1257300	656270	998910	850020
pme3163	D-Sedoheptuiose 7-phosphate	Others	606250	648820	900880	1092500	943690	1121200
pmc0066	2'-Deoxyinosine-5'-monophosphate	Nucleotides and derivatives	888560	1049000	1077600	801810	726920	705030
mws0227	L-Leucine*	Amino acids and derivatives	1135000	955150	926180	1128600	1098900	1177500

pmn001367	Protocatechuic acid-4-glucoside	Phenolic acids	942650	971830	1043600	637790	766000	821430
pme1210	L-Methionine	Amino acids and derivatives	1056600	779160	778950	985280	940050	1052800
mws0671	L-Homoserine*	Organic acids	807390	737350	772300	1387500	1111100	1098800
mws0567	4-Guanidinobutyric acid	Organic acids	676060	786650	775340	644150	703440	683730
pme0516	Inositol	Others	909810	885490	887520	750670	753740	726150
pme1436	p-Coumaric acid	Phenolic acids	712140	843930	870240	1010000	785060	822690
Zmxn001997	Isosalicylic acid O-glycoside	Phenolic acids	1227300	1586800	1321000	843520	818530	910780
pme2693	N-Acetylputrescine	Alkaloids	606510	754190	685360	625120	741130	735980
pmb2826	Citramalate	Organic acids	902370	748450	658620	996140	960500	858760
pme3337	N6-Succinyl Adenosine	Nucleotides and derivatives	670460	752490	686170	727170	748320	731930
pmb0501	Agmatine	Alkaloids	600930	661630	661550	630960	652580	704160
pmn001517	3,4,5-Trimethoxyphenyl-β-D-Glucopyranoside	Phenolic acids	769120	1072200	995770	954150	961150	998290
pme2266	Biotin	Others	566090	549890	529190	679480	728850	765420
pme3705	D-Glucoronic acid	Others	871190	574430	599390	875910	823750	943940
pmb0981	Adenosine 5'-monophosphate	Nucleotides and derivatives	801730	699760	698240	558830	679090	640140
mws0237	Anchoic Acid	Organic acids	686290	617180	667200	677540	705800	646330
Lmtn002565	1'-O-Vanilloyl-β-D-glucoside	Phenolic acids	483490	587250	546900	424750	550400	415420
HmLn000873	2-O-Galloyl-β-D-glucose	Tannins	1052000	1287200	756580	179260	214500	254940
pmp001281	LysoPC 18:1	Lipids	605400	601860	373750	676070	230510	719130
Lmtn002233	Androsin	Others	462960	644400	568960	455750	418290	416630

pme3732	Cytidine	Nucleotides and derivatives	411540	474810	505720	525740	564700	463190
mws0232	Riboflavin	Others	612410	618590	649540	653680	724320	676650
pme0281	Terephthalic acid	Phenolic acids	662730	653560	573700	561580	672770	567930
pmb0865	LysoPC 18:3(2n isomer)	Lipids	561320	550040	445110	414780	273130	365040
pme0274	6-Aminocaproic acid	Organic acids	557370	488100	661860	830880	627050	520370
pmb0764	4-Methyl-5-thiazoleethanol	Others	352070	316720	340530	459560	559640	369230
mws0752	Undecylic Acid	Lipids	555430	582750	524980	442880	450240	440410
mws0749	4-Hydroxybenzoic acid	Phenolic acids	507660	583240	496690	550070	499250	466500
mws5038	Isomaltulose*	Others	419040	399670	368770	527920	511580	485180
pme0519	D-(+)-Sucrose*	Others	373300	316590	371380	467410	432860	499670
pmn001706	2-Hydroxyoleanolic acid	Terpenoids	237480	189460	187190	50863	66874	62349
pme2746	Flavin adenine dinucleotide(FAD)	Nucleotides and derivatives	547970	474670	432400	436870	440080	474960
pmp001285	Phthalic anhydride	Phenolic acids	548400	547230	475740	309230	402240	379570
pme1109	Guanine	Nucleotides and derivatives	320370	318840	292140	373360	423250	370210
mws0376	Fumaric acid	Organic acids	451800	450530	466210	391610	412230	406720
mws4170	D-Glucose	Others	373690	397350	385830	378900	361310	387150
pme3313	D-Fructose 6-phosphate	Others	409950	281190	295480	215250	399160	412420
mws1050	O-Acetylserine	Amino acids and derivatives	231590	214830	248110	452290	502310	310570
pme1975	Malonic acid	Organic acids	386180	351120	369730	351100	311420	336790
pmb0874	LysoPE 18:2(2n isomer)	Lipids	257210	265410	131630	329570	90173	361160
mws1038	(R)-Pantetheine	Others	435770	476480	373790	291230	298830	267870

mws0230	L-(-)-Threonine*	Amino acids and derivatives	240740	284700	292720	647460	422880	444980
pmb0530	Nicotinic acid adenine dinucleotide	Nucleotides and derivatives	373860	352060	259730	282880	249670	308700
mws4134	Oxidized Glutathione	Amino acids and derivatives	319480	259980	257480	423400	404380	374090
mws2623	11-Octadecanoic acid(Vaccenic acid)	Lipids	450540	282790	310620	238550	274020	277170
mws0254	L-Histidine	Amino acids and derivatives	245570	211080	237890	383020	325040	366180
pmb3894	Di-O-methylquercetin	Flavonoids	192790	201700	201450	237110	229840	187280
Hmtp000776	4,5,6-Trihydroxy-2-cyclohexen-1-ylideneacetonitrile	Alkaloids	313230	282500	295060	295940	298980	286560
pmb0854	LysoPC 18:3	Lipids	245790	254760	194810	237830	140540	201140
pme2598	3,4-Dihydroxybenzeneacetic acid	Phenolic acids	280690	279280	268460	150190	187060	250050
mws0473	2-Methylsuccinic acid	Organic acids	366720	320740	242520	316800	301200	311500
pmn001694	9,10,13-Trihyroxy-11-octadecadienoic acid	Lipids	172540	176850	175860	207890	199600	164860
mws0133	Nicotinamide	Others	125070	122750	83534	221000	164460	128700
pmb0786	Glucosamine	Others	234070	215490	238650	269720	230130	238640
pmd0132	LysoPC 16:0(2n isomer)	Lipids	207500	201610	139750	230760	106310	240740
pmn001495	Hexadecanoic acid 2,3-dihydroxypropyl ester	Lipids	163210	171040	167000	198800	188000	161210
mws0248	Uridine	Nucleotides and derivatives	186420	175260	217240	201200	247590	188320
Rfmb320	1,2-N-Methylpipecolic acid	Amino acids and derivatives	218380	242290	221180	192070	161150	182160

pme0122	N6-Acetyl-L-lysine	Amino acids and derivatives	315360	247540	296230	266270	306740	286720
mws0258	L-Isoleucine*	Amino acids and derivatives	326890	238500	254610	276420	344240	263540
mws0668	Xanthosine	Nucleotides and derivatives	223390	198990	211220	141050	142900	134660
mws0147	3-Hydroxy-3-methyl butyric acid	Organic acids	341700	320330	324280	170560	189120	164100
Hmtn001302	Glucosyloxybenzoic acid	Phenolic acids	180390	202070	237660	156890	180300	204700
mws1333	Melibiose*	Others	142580	152820	186300	209180	149080	202420
Zmhn002422	Feruloyl glucose	Phenolic acids	237860	496010	402030	188250	261470	156760
Zmhn001926	Salicylic acid O-glycoside	Phenolic acids	171980	181620	216010	129190	161680	219340
pmb2778	9,10-EODE*	Lipids	278670	204800	198230	177520	142830	167390
mws0736	N-Glycyl-L-leucine*	Amino acids and derivatives	219240	204900	195930	177110	184320	183070
pme0490	Nicotinic acid	Others	156110	134370	136040	149670	235420	125180
mws0208	Adipic Acid	Organic acids	189710	170490	197420	181780	162880	166900
pme1014	Menaquinone (K2)	Others	155080	102450	104210	108100	80589	152900
pmb0964	Isopentenyladenine-7-N-glucoside	Nucleotides and derivatives	171680	146320	198970	146250	157650	174170
pmn001511	3-Hydroxy-5-Methylphenol-1-Oxy-β-D-Glucose	Phenolic acids	139260	147250	120870	97988	110540	81502
pmb0449	2-Aminoadipic acid (L-Homoglutamic acid)	Amino acids and derivatives	131700	126920	144000	166660	151510	133560
mws5041	Glycylisoleucine*	Amino acids and derivatives	153430	159260	160530	142900	146040	133960
pmb3099	Diethyl phosphate	Organic acids	154440	150780	180440	110330	153330	143230

pme3083	2-(Formylamino)benzoic acid	Phenolic acids	127040	139410	135480	151920	119030	151540
pme3967	2-(Dimethylamino)guanosine	Nucleotides and derivatives	123510	145670	134740	112840	122580	123440
mws0609	Guanosine 3',5'-cyclic monophosphate	Nucleotides and derivatives	142980	102870	101180	125440	86586	96725
pmb1912	10-Formyl-THF	Alkaloids	154420	141970	130220	130100	135140	116030
mws0458	Vanillin	Phenolic acids	97317	83907	101270	143830	150150	156710
pme0010	L-Serine	Amino acids and derivatives	158410	105920	136810	138440	105440	145470
pme0256	Xanthine	Nucleotides and derivatives	90597	89720	136610	124970	76782	88313
pmb2497	4-Hydroxy-3-methoxymandelate	Phenolic acids	94810	91443	115610	105750	97778	82358
pme1216	2-Picolinic acid	Organic acids	110410	79913	87948	116060	150430	101030
pme0253	N-Acetyl-L-leucine	Amino acids and derivatives	79652	86335	71095	80423	111270	75926
pmb0464	Aspartic acid di-O-glucoside	Amino acids and derivatives	94874	81821	72231	131250	149560	142690
pmb0876	LysoPE 16:0	Lipids	57451	55864	21622	111140	27804	112630
pme3961	Deoxyadenosine	Nucleotides and derivatives	97636	89809	96638	111490	103930	85236
pmb3107	Syringic acid O-glucoside	Phenolic acids	207780	260710	173940	82755	74190	92066
Smsn001839	Dihydrocornin	Terpenoids	82929	75429	65774	89410	93891	108290
mws0126	LysoPC 18:0	Lipids	84162	89638	41401	90067	17864	141100
pme2735	S-Adenosylmethionine	Amino acids and derivatives	119320	90742	99710	79460	83289	89552

mws0675	β-Nicotinamide mononucleotide	Nucleotides and derivatives	111220	132650	170850	67602	75504	70390
pme0183	2-Hydroxy-6-aminopurine	Nucleotides and derivatives	96790	83481	87428	107290	87526	91469
pme2755	N-Acetyl-D-glucosamine	Others	99738	91839	106640	107960	105580	101320
pme2651	NADP (Nicotinamide adenine dinucleotide phosphate)	Nucleotides and derivatives	108350	92247	79380	125130	108630	108690
mws1080	Galactinol*	Others	100600	127500	112730	123000	102710	91409
pmp001276	2,3-Dihydroxypropyl-9,12,15-octadecatrienoate-hexose-hexose	Lipids	115520	145170	105400	85542	82964	72883
pmf0440	4-MethoxycinnaMaldehyde	Phenolic acids	109730	102350	106860	87582	115040	96193
mws5042	Glycylphenylalanine	Amino acids and derivatives	99862	93842	87296	100780	93518	95468
mws0255	Cytosine	Nucleotides and derivatives	65968	76274	78060	83659	93726	86490
mws1060	9-(β-D-Arabinofuranosyl)hypoxanthine	Nucleotides and derivatives	96460	82905	82651	49054	61529	48453
pmn001690	3-Hydroxy-4-isopropylbenzylalcohol 3-glucoside	Phenolic acids	73970	91213	72609	77843	86150	69879
pme2559	N-Acetylaspartate	Amino acids and derivatives	67749	72420	66522	74944	82302	107160
mws2212	Caffeic acid	Phenolic acids	54126	46820	71222	112540	92711	105020
pme0001	Hesperetin 7-O-neohesperidoside(Neohesperidin)	Flavonoids	9	9	11942	9	9	9
mws0179	Chlorogenic acid methyl ester	Phenolic acids	98180	92097	94780	41907	51096	42059
Lmhp011562	1-α-Linolenoyl-glycerol*	Lipids	87046	72220	70359	70026	87657	69727

mws0884	Cyclic AMP	Nucleotides and derivatives	95623	88930	94884	83626	80249	73448
pmb0889	Punicic acid	Lipids	108030	74959	71977	73347	60030	73101
Rfmb090	13-Hydroxy-9,11-octadecadienoic acid*	Lipids	112590	77892	72937	71031	56409	76747
mws1491	Linoleic acid	Lipids	60050	67652	69903	93103	82476	83090
pmb2561	N-Acetylmethionine	Amino acids and derivatives	46137	21962	17615	3628.5	8027.5	8247.5
mws0972	5-Hydroxyhexanoic acid	Organic acids	83501	88152	75855	66434	84909	72624
pme0075	N-Acetyl-L-glutamic acid	Amino acids and derivatives	43900	51245	56177	59038	52580	81611
Lmhp012042	2-Linoleoylglycerol*	Lipids	76980	60367	57422	51683	79903	60144
pme1419	L-Methionine methyl ester	Amino acids and derivatives	80689	63642	65977	67687	76891	74034
pmn001688	9S-Hyroxy-10E,12E-octadecadienoic acid*	Lipids	102450	67788	65405	64492	52357	66022
mws0359	Pentadecanoic Acid	Lipids	75391	71231	67852	87817	91585	83556
Lmhp011388	2- γ -Linolenoyl-glycerol*	Lipids	78969	61550	60019	56747	64596	48270
pma0149	Sinapoyl malate	Phenolic acids	63260	62166	54739	84924	76069	82030
mws0520	N-Acetyl-L-tyrosine	Amino acids and derivatives	17746	19201	17932	18360	22055	17212
pmb0374	Aminopurine	Alkaloids	77836	65541	67820	69417	60712	58972
mws0191	Betaine	Alkaloids	56223	64503	69053	52088	51528	48892
pme3186	DL-Glyceraldehyde 3-phosphate	Organic acids	39534	35042	48042	76965	69708	83089
pme0295	4-Acetamidobutyric acid	Organic acids	71515	54955	55345	51077	60147	53062

mws5037	Alanylleucine	Amino acids and derivatives	65117	63809	65274	63621	89454	61828
pme1002	L-Tyramine	Amino acids and derivatives	21312	9062.2	11650	9	9	9
Lmhp112042	1-Linoleoylglycerol*	Lipids	46282	38680	42941	45380	52216	61616
pme2117	Adenosine 5'-Diphosphate	Nucleotides and derivatives	65452	55702	45242	59478	68953	54441
mws0847	1-Methyladenine	Nucleotides and derivatives	56091	39308	54368	46847	53424	55207
mws0146	Nicotinic Acid Methyl Ester(Methyl Nicotinate)	Alkaloids	40578	41781	42109	49473	72445	60544
mws0001	L-Asparagine	Amino acids and derivatives	43195	38466	48689	31366	46640	50266
mws1200	Trans-4-Hydroxycinnamic Acid Methyl Ester	Phenolic acids	35814	48280	59491	56802	44703	66189
mws0341	(S)-(-)-2-Hydroxyisocaproic acid	Organic acids	35778	33799	46204	38559	48035	30993
pme3388	H-HomoArg-OH	Amino acids and derivatives	35372	37658	35619	50362	53620	76568
pme1286	S-(5'-Adenosy)-L-homocysteine	Amino acids and derivatives	41274	33640	37111	38864	37430	52859
pme2529	1,5-Anhydro-D-glucitol	Others	62846	44954	38636	22620	30608	45823
pmf0359	Skimmin	Lignans and Coumarins	59379	61761	47899	34042	27911	28479
pmb0881	LysoPE 18:2	Lipids	23718	15910	8449.6	47187	13400	53599
pme0170	N-α-Acetyl-L-arginine	Amino acids and derivatives	29830	32282	32977	39638	37514	33337

pme0278	2,6-Diaminooimelic acid	Amino acids and derivatives	35781	32465	35909	63327	53225	64252
pmb2406	LysoPC 17:0	Lipids	41244	39148	25510	41249	11242	41048
Lmtn002796	Aromadendrin 7-glucoside	Others	47000	34779	45311	42739	41980	39378
pme2596	4-Pyridoxic acid	Others	44249	46233	45986	50853	49579	51872
mws0629	Asp-phe	Amino acids and derivatives	35204	36495	40028	42971	41297	42601
pmn001606	Eicosenoic acid	Lipids	39340	34591	31823	49754	58486	52982
pme3382	N-Acetylthreonine	Amino acids and derivatives	30576	32202	33008	39441	30685	32777
Lmyn000239	Cordycepic acid*	Others	40929	41302	53796	36223	31810	44675
mws0193	L-Homocitrulline	Amino acids and derivatives	62060	46470	55885	50764	49131	49785
pme1184	Deoxyguanosine	Nucleotides and derivatives	34782	32532	33288	27778	33207	32763
pmp001275	3-Hydroxypropyl palmitate glc-glucosamine	Alkaloids	45940	48855	47145	34236	35639	31199
pmn001668	Apigenin-3-O-α-L-rhamnoside	Flavonoids	28165	28929	32016	27768	28580	36026
Zmhn002227	Sinapic acid-glycoside	Phenolic acids	35001	41955	39716	42133	31073	32577
mws4176	Alanylphenylalanine	Amino acids and derivatives	36563	36636	34879	30411	36566	27754
pmp001282	Propyl2-(trimethylammonio)ethyl phosphate	Others	39662	36326	21627	27859	8411.6	26339
pmb0197	N2-methylguanosine	Nucleotides and derivatives	25075	28755	26909	38851	47123	32704
Lmdp003090	Dihydroquercetin(Taxifolin)	Flavonoids	31430	29738	27252	25175	31102	26029

Lmhn002683	p-Coumaroylcaffeoyltartaric acid	Phenolic acids	35119	37564	26639	20516	15718	13428
pme1194	Deoxycytidine	Nucleotides and derivatives	33391	30370	26639	28413	28979	28821
pme3174	Cytidine 5'-monophosphate(Cytidylic acid)	Nucleotides and derivatives	19646	21798	22210	33567	24613	20808
YC512118	Octadecenoic amide	Others	35180	15721	34934	31986	8316.3	12411
pme0264	Thymidine	Nucleotides and derivatives	32963	22219	26401	23505	32245	29889
mws1499	D-(-)-Arabinose	Others	20466	36403	29885	32031	29758	34138
pmb2792	13-HOTrE(r)	Lipids	21283	28551	27979	25917	20638	17596
pmb0856	LysoPE 18:1(2n isomer)	Lipids	27972	25954	15500	28422	11843	30857
pmb2363	MAG(18:1)	Lipids	32952	42995	30534	23330	24762	24001
pme1021	D-(+)-Glucono-1,5-lactone	Others	22425	22460	35228	20779	21014	16529
pmb2653	D(+)-Melezitose O-rhamnoside	Others	15971	28076	29332	34818	22956	16778
pme3968	7-Methylguanine	Nucleotides and derivatives	16940	17825	18517	27722	32589	21548
Lmhp008589	LysoPE 18:3(2n isomer)	Lipids	16152	17795	10830	18627	5975.9	22848
mws0177	2-Furanoic acid	Organic acids	9236.2	9134.1	7626.6	21683	27232	16723
pmn001336	Aloeemodin-8-O-D-glucopyranoside	Quinones	23888	30893	26011	31247	28531	25120
pme0195	L-Cysteine	Amino acids and derivatives	30875	30443	35936	21848	26193	20038
pmn001352	6-Hydroxyrumicin-8-O-D-glucopyranoside	Quinones	33318	28616	29740	16413	14227	19310
Lmdp003994	Wistin (6,4'-Dimethoxyisoflavone-7-glucoside)	Flavonoids	21330	24943	20413	13600	16163	13654

pmb2654	Anthranilate O-hexosyl-O-hexoside	Phenolic acids	20355	19446	25235	32051	27080	30595
pme0241	Benzoic acid	Phenolic acids	25377	28511	21464	21982	22987	20485
mws0011	Syringin	Phenolic acids	30753	48068	63460	15886	8292.8	11262
Lmtn003598	3-Prenyl-4-O-β-D-glucopyranosyloxy-4-hydroxyl-benzoic acid	Phenolic acids	28171	28675	29777	25663	40200	26783
pmb0801	4-Pyridoxic acid O-hexoside	Others	19616	25545	23308	23939	18313	23953
Hmpp003242	Isorhamnetin-3-O-β-D-glucoside	Flavonoids	21683	27861	17994	17556	17910	14698
pme2527	L-Ornithine	Amino acids and derivatives	17419	15760	15656	32370	28737	34813
pmp001270	LysoPC 16:1	Lipids	20567	21254	18195	15210	7953.8	14116
pmn001681	1-(4-Methoxyphenyl)-1-propanol	Phenolic acids	17620	15851	18736	15732	16533	15685
Lmhn002926	p-Coumaroylmalic acid	Phenolic acids	19551	25505	24941	27909	22023	28944
Lmhp009590	LysoPC 17:1	Lipids	23488	19541	15399	18879	9901.2	18330
pmp001312	6-Hydroxykaempferol-3,7,6-O-triglycoside	Flavonoids	15991	17759	13340	11733	11935	14557
pme0008	L-Citrulline	Amino acids and derivatives	10972	8463.4	13293	33336	18971	20402
YC512119	Phytosphingosine	Others	16578	18780	14681	17514	16166	19852
mws0242	SubericAcid	Organic acids	12612	16811	13507	22766	16538	13164
mws1421	α-Viniferin	Others	9502.8	6797	5292.9	9	11194	9
pme0181	1-Methylhistidine	Amino acids and derivatives	16751	18496	17076	8910.9	18778	16900
Lmhn003074	Feruloylmalic acid	Phenolic acids	30301	41023	43119	27863	14282	16516
pme1730	D-Erythronolactone	Organic acids	16463	15530	17574	14595	14060	11995

mws1383	Lumichrome	Alkaloids	12701	14798	11250	20367	13097	18278
mws2523	Trehalose 6-phosphate	Others	12709	12060	10751	13484	13262	12425
mws0852	Methotrexate	Nucleotides and derivatives	10699	10938	10119	12461	12070	13994
pmb2260	LysoPC 15:1	Lipids	14958	14958	13324	14258	12256	13337
pme0033	Hypoxanthine	Nucleotides and derivatives	12341	14753	10903	10783	18204	10322
mws0124	N-(3-Indolylacetyl)-L-alanine	Amino acids and derivatives	8710.7	9291.4	8493.1	7765.3	6699.6	6918.5
pmb2804	13-HPODE	Lipids	16771	13278	13519	12394	12514	12455
mws0361	Palmitoleic Acid	Lipids	10080	8617.1	10421	12486	15749	13691
mws0572	5-Methylcytosine	Nucleotides and derivatives	11906	16737	12424	12796	12762	15165
pme0120	5-Aminovaleric acid*	Amino acids and derivatives	8451	6424.6	7990.7	17172	17286	16618
pme1738	3-Carbamyl-1-methylpyridinium(1-Methylnicotinamide)	Others	12386	10976	9074.5	15383	11690	14835
pmb2657	Argininosuccinic acid	Organic acids	9801.5	9092.9	7572.5	11244	10856	14649
mws0981	Isoxanthopterin	Nucleotides and derivatives	15321	14369	15924	12269	9251.6	10851
pmd0160	LysoPE 16:0(2n isomer)	Lipids	7026.9	7423.9	3419.6	14042	2945.7	12158
pme1187	5-Methyluridine	Nucleotides and derivatives	15030	12135	11399	8733.6	13183	13160
mws1589	Panose*	Others	8302.3	8542.6	8420.3	9305.1	8738.4	11786
Hmlp001371	Cyclo(Tyr-Ala)	Amino acids and derivatives	16145	13701	13579	6325.9	7994.8	7787.6

pmb2857	L-Glutamic acid O-glycoside	Amino acids and derivatives	9	9	2709.1	11946	19188	9
mws5035	Leucylphenylalanine	Amino acids and derivatives	12472	11679	11356	9256.1	16909	9961.7
pmb3075	3-O-p-Coumaroylshikimic acid	Phenolic acids	6825.3	9351.3	10247	9509.6	10698	10740
Lmhp008718	LysoPC 17:2	Lipids	10922	8950.5	7768.8	8481.5	4942.3	7498.1
mws0057	Eriodictyol 7-O-glucoside	Flavonoids	6451.3	8261.7	6842.8	4679	5387.3	4608.6
pmb2799	12,13-EODE	Lipids	9456.7	6894.5	6012.9	8094.9	10123	8308.2
Hmjn008136	Camaldulenic acid	Terpenoids	9	9	9	9	9	9
pmb0962	Lysine butyrate	Amino acids and derivatives	13193	7778.4	18048	9699.2	13036	13825
mws1078	Anthranilic Acid	Phenolic acids	11791	10311	8316.5	5781.3	7653.7	6815
pmb1096	Indole	Alkaloids	8287	9395.1	8097.8	10173	11770	9449.8
mws2218	Caffeine	Alkaloids	9	9	9	9	17534	6234.7
mws1420	ε-Viniferin	Others	5512.7	8087.5	3145.6	9	9	9
pmd0136	LysoPC 18:0(2n isomer)	Lipids	7354.5	5688.9	3655.8	6556.7	1934.8	10908
pme2237	Dulcitol*	Others	10226	10847	9338.8	8553	8162.7	4552.4
pmf0297	1-Eicosanol	Lipids	8197.5	9602.9	5738.5	11852	9444.7	6082.1
Hmbn002174	4-Hydroxyacetophenone	Phenolic acids	5047.5	6123.9	6986.8	8647.3	8503.5	9047.2
Hmcp002268	Limocitrin 3-rhamnoside	Flavonoids	3903.8	4798.1	4923.7	3754	4830.7	2461.8
mws0596	3-Hydroxyanthranilic acid	Organic acids	8150	8242.9	9	8929.1	8738.2	7755.6
pmb2791	9-HpOTrE	Lipids	8382.6	7101	8247.8	6291.6	7364.5	6466.3
mws1212	Methyl ferulate	Phenolic acids	5885.3	8613.7	9950.9	13102	6100.1	11800
pmb2640	Lauric acid	Lipids	7455.7	6509.3	5559.8	6496.1	5745.1	5612.4
mws0289	LysoPE 18:1	Lipids	9	9	9	6013.9	9	6149.3

pme0500	D-(+)-Melezitose*	Others	2715.1	4134.4	2997.6	6257.6	5345.1	7190.7
pmn001691	9,12,13-Trihyroxy-10,15-octadecadienoic acid	Lipids	3672.2	5119.9	3752.1	4657.8	4225.8	3475.7
pmn001695	Trihydroxycinnamoylquinic acid	Phenolic acids	16000	21300	11703	9	9	9
mws0120	Choline alfoscerate	Lipids	5512.2	2645.2	1735.5	9	9	3206.2
Lmhp008801	LysoPE 18:3	Lipids	9	9	9	6284.1	9	4722.2
pme2890	L-Homocystine	Amino acids and derivatives	5884	4080.6	6138.9	4224.2	3710.6	3953.7
Lmhp009129	LysoPC 15:0	Lipids	4919.1	4952.1	3530.4	3866.5	2227	4999.7
mws0636	Phe-Phe	Amino acids and derivatives	5449.5	4582.7	4464.8	4162.6	4736.3	5950.7
pmb2786	9-HOTrE	Lipids	4743.7	4004.1	3241.7	3439.5	2375.6	2033.6
pmb1283	L-Glutaminyl-L-valyl-L-valyl-L-cysteine	Amino acids and derivatives	3084.7	4965.1	4143.2	9	9	9

(Control)

Index	Compounds	Class I	Control_1	Control_2	Control_3
mws0470	Methylmalonic acid*	Organic acids	36326000	35205000	41167000
mws0192	Succinic acid*	Organic acids	33646000	32540000	37590000
pme0230	Adenosine	Nucleotides and derivatives	21156000	24476000	22351000
pme0021	L-Phenylalanine	Amino acids and derivatives	20915000	21114000	21251000
mws0277	Kinic acid	Organic acids	15890000	17900000	19026000
pmb0789	Pyridoxine-5'-O-glucoside	Others	15091000	14242000	17329000

mws0216	Trans-4-Hydroxy-L-proline	Amino acids and derivatives	20417000	22638000	22156000	
mws0281	Citric Acid	Organic acids	14579000	16170000	16531000	
pmc0274	6-Methylmercaptopurine	Nucleotides and derivatives	15447000	15157000	15643000	
Rfmb324	Trigonelline	Alkaloids	11262000	12220000	11752000	
pme0006	L-Proline	Amino acids and derivatives	12139000	11457000	11253000	
pmn001578	Hexadecanoic acid	Lipids	7954000	9026600	6992100	
mws0119	Myristic Acid	Lipids	7553200	8689200	6319200	
pmp001287	N-Benzylmethylene isomethylamine	Alkaloids	7768500	7846500	7773400	
pme1474	5'-Deoxy-5'-(methylthio)adenosine	Nucleotides and derivatives	7729500	7447800	6413800	
mws1489	Stearic Acid	Lipids	5581900	6710000	5427600	
mws0256	L-Valine*	Amino acids and derivatives	7725000	7415000	7517000	
Zmhn001970	Piceid	Others	3182700	1895700	2551200	
pmb0855	LysoPC 16:0	Lipids	6355700	1668700	4987900	
mws0250	L-(-)-Tyrosine	Amino acids and derivatives	5574100	5046500	5417300	
pme1383	Pyridoxine	Others	3042600	3320300	4155400	
mws0366	γ-Linolenic Acid	Lipids	6965700	11364000	4964100	
pmp001198	6-Deoxyfagomine	Alkaloids	5145100	4736200	4614800	
pmb3101	2-Isopropylmalate	Organic acids	3537200	3545900	3006600	
Hmsn000210	Methyl 7,10-hexadecadienoate	Lipids	3307800	3451900	3847600	

pme2380	A-Ketoglutaric acid	Organic acids	2908600	3243600	3016200	
pma3101	Nicotinate D-ribonucleoside	Others	3439500	3545800	3222400	
mws1337	D-Pantothenic Acid	Others	3020200	3277400	3410400	
pmp001273	LysoPC 18:2	Lipids	3871500	1155700	3040900	
pmb2922	Uridine 5'-diphospho-D-glucose	Nucleotides and derivatives	3826500	3978600	3915500	
pme2914	3-Hydroxy-3-methylpentane-1,5-dioic acid	Amino acids and derivatives	2601800	2319100	2634800	
pme3011	γ-Aminobutyric acid*	Organic acids	3390600	3019000	3030000	
mws0282	L-Tryptophan	Amino acids and derivatives	3089000	2591400	2807200	
Zmhn001414	Resveratrol-O-diglucoside	Others	2468000	3190600	3136900	
pmn001419	1-O-[(E)-p-Cumaroyl]-β-D-glucopyranose	Phenolic acids	1903600	1397500	2812200	
pme0040	Adenine	Nucleotides and derivatives	2795300	3096100	2751200	
pmb1650	Octadeca-11E,13E,15Z-trienoic acid	Lipids	2977500	2532200	2569000	
pmn001420	1-O-[(E)-Caffeoyl]-β-D-glucopyranose	Phenolic acids	2117300	2110100	2788800	
pme3017	2-Aminoisobutyric acid*	Amino acids and derivatives	2468000	2555600	2366700	
pme0014	L-Glutamic acid	Amino acids and derivatives	2208300	2356000	2442900	
Rfmb319	Pipecolic acid	Amino acids and derivatives	1765000	2090200	1638300	
pme0193	L-Glutamine	Amino acids and derivatives	2590300	2416300	2539200	
mws5040	Turanose	Others	2120300	1850100	2061400	

pme1178	Guanosine	Nucleotides and derivatives	1976500	2199300	2299200
pmb3081	Glucarate O-Phosphoric acid	Others	2211200	1698300	2239200
pme0026	L-(+)-Lysine	Amino acids and derivatives	2317900	2216700	2595200
pme3184	2'-Deoxyadenosine-5'-monophosphate	Nucleotides and derivatives	1501600	1612400	2125500
mws0260	L-(+)-Arginine	Amino acids and derivatives	2379400	2194600	2103400
pme3033	N,N-Dimethylglycine	Amino acids and derivatives	2049400	2009100	1889400
pme3096	Aminomalonic acid	Organic acids	1422100	1449000	1499600
mws0219	L-AsparticAcid	Amino acids and derivatives	1410100	1442000	1560400
pmp001086	5-Hydroxymethylfurfural	Phenolic acids	1543000	1483500	1479100
mws0275	L-(-)-Malic acid	Organic acids	1344800	1330600	1520100
Lmhp010190	LysoPC 18:1(2n isomer)	Lipids	1331300	305100	923510
mws0866	D-Glucose 6-phosphate	Others	1295100	1173500	1460600
mws0628	4-Hydroxybenzaldehyde	Phenolic acids	1165300	1035000	1094100
pme0534	Gluconic acid	Others	1252000	1505200	1261100
mws0983	N-Oleoylethanolamine	Others	868500	1313400	1059900
pme3163	D-Sedoheptuiose 7-phosphate	Others	1151900	869720	930730
pmc0066	2'-Deoxyinosine-5'-monophosphate	Nucleotides and derivatives	938010	863900	636010
mws0227	L-Leucine*	Amino acids and derivatives	1098800	997280	1078100

pmn001367	Protocatechuic acid-4-glucoside	Phenolic acids	609860	692560	641480
pme1210	L-Methionine	Amino acids and derivatives	1021500	933720	975590
mws0671	L-Homoserine*	Organic acids	835650	876210	924500
mws0567	4-Guanidinobutyric acid	Organic acids	1202800	1037400	1344100
pme0516	Inositol	Others	727620	726860	798560
pme1436	p-Coumaric acid	Phenolic acids	1079600	760850	1061800
Zmxn001997	Isosalicylic acid O-glycoside	Phenolic acids	543110	454430	653430
pme2693	N-Acetylputrescine	Alkaloids	715030	662860	835430
pmb2826	Citramalate	Organic acids	745490	781670	833370
pme3337	N6-Succinyl Adenosine	Nucleotides and derivatives	811410	758960	825430
pmb0501	Agmatine	Alkaloids	685480	601820	822390
pmn001517	3,4,5-Trimethoxyphenyl- β -D-Glucopyranoside	Phenolic acids	639230	678840	949230
pme2266	Biotin	Others	664440	800790	851170
pme3705	D-Glucoronic acid	Others	889960	1224200	1028500
pmb0981	Adenosine 5'-monophosphate	Nucleotides and derivatives	548160	614380	608670
mws0237	Anchoic Acid	Organic acids	825110	775980	675460
Lmtn002565	1'-O-Vanilloyl- β -D-glucoside	Phenolic acids	478200	575960	668610
HmLn000873	2-O-Galloyl- β -D-glucose	Tannins	493080	419880	408950
pmp001281	LysoPC 18:1	Lipids	678910	115500	512930
Lmtn002233	Androsin	Others	607280	524110	709490
pme3732	Cytidine	Nucleotides and derivatives	823720	788590	716320

mws0232	Riboflavin	Others	525390	562180	549390
pme0281	Terephthalic acid	Phenolic acids	573740	559620	567750
pmb0865	LysoPC 18:3(2n isomer)	Lipids	1236600	388440	436460
pme0274	6-Aminocaproic acid	Organic acids	703480	681120	641470
pmb0764	4-Methyl-5-thiazoleethanol	Others	502220	563690	385780
mws0752	Undecylic Acid	Lipids	518900	503070	446070
mws0749	4-Hydroxybenzoic acid	Phenolic acids	530310	406000	460580
mws5038	Isomaltulose*	Others	353820	546390	311500
pme0519	D-(+)-Sucrose*	Others	394750	501030	632100
pmn001706	2-Hydroxyoleanolic acid	Terpenoids	762660	849100	551410
pme2746	Flavin adenine dinucleotide(FAD)	Nucleotides and derivatives	417400	418960	459760
pmp001285	Phthalic anhydride	Phenolic acids	518600	433930	435890
pme1109	Guanine	Nucleotides and derivatives	579380	589870	482340
mws0376	Fumaric acid	Organic acids	389050	315420	349200
mws4170	D-Glucose	Others	380080	344000	472150
pme3313	D-Fructose 6-phosphate	Others	255240	317760	329770
mws1050	O-Acetylserine	Amino acids and derivatives	498560	571930	260110
pme1975	Malonic acid	Organic acids	339210	455110	410110
pmb0874	LysoPE 18:2(2n isomer)	Lipids	366110	115700	160160
mws1038	(R)-Pantetheine	Others	323980	292970	257510
mws0230	L-(-)-Threonine*	Amino acids and derivatives	366440	327550	350380

pmb0530	Nicotinic acid adenine dinucleotide	Nucleotides and derivatives	373390	344650	261660
mws4134	Oxidized Glutathione	Amino acids and derivatives	293370	427490	382180
mws2623	11-Octadecanoic acid(Vaccenic acid)	Lipids	202800	308370	174350
mws0254	L-Histidine	Amino acids and derivatives	309880	287680	326960
pmb3894	Di-O-methylquercetin	Flavonoids	380060	289250	242980
Hmtp000776	4,5,6-Trihydroxy-2-cyclohexen-1-ylideneacetonitrile	Alkaloids	294250	297530	290740
pmb0854	LysoPC 18:3	Lipids	472870	129840	240700
pme2598	3,4-Dihydroxybenzeneacetic acid	Phenolic acids	157540	152260	134450
mws0473	2-Methylsuccinic acid	Organic acids	161990	226460	271670
pmn001694	9,10,13-Trihyroxy-11-octadecadienoic acid	Lipids	334250	253310	208910
mws0133	Nicotinamide	Others	310790	227060	128980
pmb0786	Glucosamine	Others	226170	231000	233690
pmd0132	LysoPC 16:0(2n isomer)	Lipids	275700	83944	228150
pmn001495	Hexadecanoic acid 2,3-dihydroxypropyl ester	Lipids	333480	239040	204950
mws0248	Uridine	Nucleotides and derivatives	328290	317980	249040
Rfmb320	1,2-N-Methylpipecolic acid	Amino acids and derivatives	651750	224570	205330
pme0122	N6-Acetyl-L-lysine	Amino acids and derivatives	220160	256630	232720
mws0258	L-Isoleucine*	Amino acids and derivatives	265970	277550	260850

mws0668	Xanthosine	Nucleotides and derivatives	195440	179840	202840
mws0147	3-Hydroxy-3-methyl butyric acid	Organic acids	155740	151970	165630
Hmtn001302	Glucosyloxybenzoic acid	Phenolic acids	161440	147100	171060
mws1333	Melibiose*	Others	206490	188630	242860
Zmhn002422	Feruloyl glucose	Phenolic acids	116990	85344	169160
Zmhn001926	Salicylic acid O-glycoside	Phenolic acids	153060	145710	169730
pmb2778	9,10-EODE*	Lipids	175220	195410	122190
mws0736	N-Glycyl-L-leucine*	Amino acids and derivatives	230540	216970	221190
pme0490	Nicotinic acid	Others	206520	227150	157080
mws0208	Adipic Acid	Organic acids	196140	187050	167160
pme1014	Menaquinone (K2)	Others	213680	166090	198090
pmb0964	Isopentenyladenine-7-N-glucoside	Nucleotides and derivatives	204550	170440	147980
pmn001511	3-Hydroxy-5-Methylphenol-1-Oxy-β-D-Glucose	Phenolic acids	121090	124190	125850
pmb0449	2-Aminoadipic acid (L-Homoglutamic acid)	Amino acids and derivatives	204730	240600	127780
mws5041	Glycylisoleucine*	Amino acids and derivatives	166470	166970	178300
pmb3099	Diethyl phosphate	Organic acids	180820	174560	127390
pme3083	2-(Formylamino)benzoic acid	Phenolic acids	155040	121610	161700
pme3967	2-(Dimethylamino)guanosine	Nucleotides and derivatives	154480	158490	179710

mws0609	Guanosine 3',5'-cyclic monophosphate	Nucleotides and derivatives	127960	90958	108920
pmb1912	10-Formyl-THF	Alkaloids	147070	130130	146160
mws0458	Vanillin	Phenolic acids	156410	148300	180430
pme0010	L-Serine	Amino acids and derivatives	94721	150320	101090
pme0256	Xanthine	Nucleotides and derivatives	185210	256270	133040
pmb2497	4-Hydroxy-3-methoxymandelate	Phenolic acids	113140	118410	134540
pme1216	2-Picolinic acid	Organic acids	174730	167050	115310
pme0253	N-Acetyl-L-leucine	Amino acids and derivatives	81026	63002	51786
pmb0464	Aspartic acid di-O-glucoside	Amino acids and derivatives	142200	138990	115310
pmb0876	LysoPE 16:0	Lipids	116560	32957	41663
pme3961	Deoxyadenosine	Nucleotides and derivatives	131770	131300	124480
pmb3107	Syringic acid O-glucoside	Phenolic acids	105300	102000	96808
Smsn001839	Dihydrocornin	Terpenoids	77193	92248	87308
mws0126	LysoPC 18:0	Lipids	137610	9445.7	95543
pme2735	S-Adenosylmethionine	Amino acids and derivatives	88966	85855	87887
mws0675	β-Nicotinamide mononucleotide	Nucleotides and derivatives	96578	120860	92122
pme0183	2-Hydroxy-6-aminopurine	Nucleotides and derivatives	163720	151010	104610

pme2755	N-Acetyl-D-glucosamine	Others	98387	113410	109920
pme2651	NADP (Nicotinamide adenine dinucleotide phosphate)	Nucleotides and derivatives	89006	88712	102100
mws1080	Galactinol*	Others	56704	84629	92548
pmp001276	2,3-Dihydroxypropyl-9,12,15-octadecatrienoate-hexose-hexose	Lipids	75538	78346	83196
pmf0440	4-MethoxycinnaMaldehyde	Phenolic acids	116390	115960	93173
mws5042	Glycylphenylalanine	Amino acids and derivatives	121920	106770	129790
mws0255	Cytosine	Nucleotides and derivatives	137080	148290	105700
mws1060	9-(β-D-Arabinofuranosyl)hypoxanthine	Nucleotides and derivatives	81956	83782	92844
pmn001690	3-Hydroxy-4-isopropylbenzylalcohol 3-glucoside	Phenolic acids	50456	55355	60244
pme2559	N-Acetylaspartate	Amino acids and derivatives	55860	53393	72098
mws2212	Caffeic acid	Phenolic acids	98317	74879	113270
pme0001	Hesperetin 7-O-neohesperidoside(Neohesperidin)	Flavonoids	1363000	9	9
mws0179	Chlorogenic acid methyl ester	Phenolic acids	57733	69477	88976
Lmhp011562	1-α-Linolenoyl-glycerol*	Lipids	89466	183230	76233
mws0884	Cyclic AMP	Nucleotides and derivatives	82345	86775	79310
pmb0889	Punicic acid	Lipids	72600	95991	51678
Rfmb090	13-Hydroxy-9,11-octadecadienoic acid*	Lipids	63512	96807	53154

mws1491	Linoleic acid	Lipids	82138	113850	82229
pmb2561	N-Acetylmethionine	Amino acids and derivatives	157550	189000	267820
mws0972	5-Hydroxyhexanoic acid	Organic acids	61662	49213	90443
pme0075	N-Acetyl-L-glutamic acid	Amino acids and derivatives	52732	60307	65095
Lmhp012042	2-Linoleoylglycerol*	Lipids	66971	155790	61424
pme1419	L-Methionine methyl ester	Amino acids and derivatives	66136	82924	80958
pmn001688	9S-Hyroxy-10E,12E-octadecadienoic acid*	Lipids	58628	86928	46258
mws0359	Pentadecanoic Acid	Lipids	67517	68793	57992
Lmhp011388	2- γ -Linolenoyl-glycerol*	Lipids	59993	158150	51943
pma0149	Sinapoyl malate	Phenolic acids	71557	62833	68133
mws0520	N-Acetyl-L-tyrosine	Amino acids and derivatives	14211	12708	15930
pmb0374	Aminopurine	Alkaloids	59557	76353	65384
mws0191	Betaine	Alkaloids	84419	59604	74615
pme3186	DL-Glyceraldehyde 3-phosphate	Organic acids	61908	77638	66712
pme0295	4-Acetamidobutyric acid	Organic acids	51173	57201	29808
mws5037	Alanylleucine	Amino acids and derivatives	56176	102370	75620
pme1002	L-Tyramine	Amino acids and derivatives	539910	60946	107350
Lmhp112042	1-Linoleoylglycerol*	Lipids	55161	118270	59908

pme2117	Adenosine 5'-Diphosphate	Nucleotides and derivatives	42521	47406	56220
mws0847	1-Methyladenine	Nucleotides and derivatives	50866	60902	59874
mws0146	Nicotinic Acid Methyl Ester(Methyl Nicotinate)	Alkaloids	33853	41937	45071
mws0001	L-Asparagine	Amino acids and derivatives	57801	44274	61758
mws1200	Trans-4-Hydroxycinnamic Acid Methyl Ester	Phenolic acids	62132	54154	92011
mws0341	(S)-(-)-2-Hydroxyisocaproic acid	Organic acids	80906	34600	53493
pme3388	H-HomoArg-OH	Amino acids and derivatives	57915	55734	59746
pme1286	S-(5'-Adenosy)-L-homocysteine	Amino acids and derivatives	46115	54191	51898
pme2529	1,5-Anhydro-D-glucitol	Others	35317	41509	44090
pmf0359	Skimmin	Lignans and Coumarins	26740	17446	28094
pmb0881	LysoPE 18:2	Lipids	55245	18472	15236
pme0170	N-α-Acetyl-L-arginine	Amino acids and derivatives	34547	29194	28689
pme0278	2,6-Diaminooimelic acid	Amino acids and derivatives	49844	49003	55801
pmb2406	LysoPC 17:0	Lipids	56693	7159.3	41461
Lmtn002796	Aromadendrin 7-glucoside	Others	20133	31827	37283
pme2596	4-Pyridoxic acid	Others	44035	44300	42364
mws0629	Asp-phe	Amino acids and derivatives	46563	48048	46014

pmn001606	Eicosenoic acid	Lipids	37631	57218	33578
pme3382	N-Acetylthreonine	Amino acids and derivatives	34831	36361	44286
Lmyn000239	Cordycepic acid*	Others	40461	35829	47714
mws0193	L-Homocitrulline	Amino acids and derivatives	34385	40581	40426
pme1184	Deoxyguanosine	Nucleotides and derivatives	33849	37546	37851
pmp001275	3-Hydroxypropyl palmitate glc-glucosamine	Alkaloids	34830	41930	41353
pmn001668	Apigenin-3-O-α-L-rhamnoside	Flavonoids	36892	44079	36617
Zmhn002227	Sinapic acid-glycoside	Phenolic acids	25490	33164	28466
mws4176	Alanylphenylalanine	Amino acids and derivatives	40474	44503	42791
pmp001282	Propyl2-(trimethylammonio)ethyl phosphate	Others	27658	4355	21088
pmb0197	N2-methylguanosine	Nucleotides and derivatives	42563	42886	47766
Lmdp003090	Dihydroquercetin(Taxifolin)	Flavonoids	23156	25494	25343
Lmhn002683	p-Coumaroylcaffeoyltartaric acid	Phenolic acids	11902	11521	10034
pme1194	Deoxycytidine	Nucleotides and derivatives	22921	33137	32720
pme3174	Cytidine 5'-monophosphate(Cytidylic acid)	Nucleotides and derivatives	32889	29554	21765
YC512118	Octadecenoic amide	Others	26723	25878	13517
pme0264	Thymidine	Nucleotides and derivatives	27445	30207	30714
mws1499	D-(-)-Arabinose	Others	30675	30940	39518

pmb2792	13-HOTrE(r)	Lipids	23942	48677	13668
pmb0856	LysoPE 18:1(2n isomer)	Lipids	31425	8715	11754
pmb2363	MAG(18:1)	Lipids	23748	22720	33766
pme1021	D-(+)-Glucono-1,5-lactone	Others	37454	30565	22946
pmb2653	D(+)-Melezitose O-rhamnoside	Others	13677	18388	15681
pme3968	7-Methylguanine	Nucleotides and derivatives	30968	27164	25511
Lmhp008589	LysoPE 18:3(2n isomer)	Lipids	77651	6760.8	14479
mws0177	2-Furanoic acid	Organic acids	25073	32558	15154
pmn001336	Aloeemodin-8-O-D-glucopyranoside	Quinones	29749	24828	29192
pme0195	L-Cysteine	Amino acids and derivatives	28053	17745	26664
pmn001352	6-Hydroxyrumicin-8-O-D-glucopyranoside	Quinones	12730	15851	17007
Lmdp003994	Wistin (6,4'-Dimethoxyisoflavone-7-glucoside)	Flavonoids	18631	19795	23461
pmb2654	Anthranilate O-hexosyl-O-hexoside	Phenolic acids	20468	24170	25461
pme0241	Benzoic acid	Phenolic acids	27534	23964	21289
mws0011	Syringin	Phenolic acids	9918.9	13211	13606
Lmtn003598	3-Prenyl-4-O-β-D-glucopyranosyloxy-4-hydroxyl-benzoic acid	Phenolic acids	17372	14218	20400
pmb0801	4-Pyridoxic acid O-hexoside	Others	21894	25239	21347
Hmpp003242	Isorhamnetin-3-O-β-D-glucoside	Flavonoids	12618	14069	19677
pme2527	L-Ornithine	Amino acids and derivatives	24395	21182	27463
pmp001270	LysoPC 16:1	Lipids	22555	8612.6	18140
pmn001681	1-(4-Methoxyphenyl)-1-propanol	Phenolic acids	18031	21975	16530

Lmhn002926	p-Coumaroylmalic acid	Phenolic acids	20041	10377	19098
Lmhp009590	LysoPC 17:1	Lipids	26155	8391.7	18971
pmp001312	6-Hydroxykaempferol-3,7,6-O-triglycoside	Flavonoids	15880	19789	18322
pme0008	L-Citrulline	Amino acids and derivatives	23616	17219	20275
YC512119	Phytosphingosine	Others	19611	17272	16602
mws0242	SubericAcid	Organic acids	23600	19650	21166
mws1421	α-Viniferin	Others	24635	15778	21653
pme0181	1-Methylhistidine	Amino acids and derivatives	17184	23333	17375
Lmhn003074	Feruloylmalic acid	Phenolic acids	10669	6012.8	9
pme1730	D-Erythronolactone	Organic acids	14537	15002	15266
mws1383	Lumichrome	Alkaloids	10826	20016	12224
mws2523	Trehalose 6-phosphate	Others	12272	16127	17045
mws0852	Methotrexate	Nucleotides and derivatives	16100	15278	16981
pmb2260	LysoPC 15:1	Lipids	16097	12950	15395
pme0033	Hypoxanthine	Nucleotides and derivatives	15486	15826	15228
mws0124	N-(3-Indolylacetyl)-L-alanine	Amino acids and derivatives	3907.2	4345.5	5095.3
pmb2804	13-HPODE	Lipids	11367	17149	10102
mws0361	Palmitoleic Acid	Lipids	19026	14073	13223
mws0572	5-Methylcytosine	Nucleotides and derivatives	18091	14376	18979

pme0120	5-Aminovaleric acid*	Amino acids and derivatives	10398	18014	16823
pme1738	3-Carbamyl-1-methylpyridinium(1-Methylnicotinamide)	Others	8851.7	15680	16823
pmb2657	Argininosuccinic acid	Organic acids	9959.1	12133	13019
mws0981	Isoxanthopterin	Nucleotides and derivatives	9201.1	11049	12267
pmd0160	LysoPE 16:0(2n isomer)	Lipids	12539	2955.9	4853
pme1187	5-Methyluridine	Nucleotides and derivatives	7402.9	7688.5	12647
mws1589	Panose*	Others	7860.1	8994	13050
Hmlp001371	Cyclo(Tyr-Ala)	Amino acids and derivatives	9924.4	6902.6	11148
pmb2857	L-Glutamic acid O-glycoside	Amino acids and derivatives	25426	28729	9
mws5035	Leucylphenylalanine	Amino acids and derivatives	9997	18029	9326
pmb3075	3-O-p-Coumaroylshikimic acid	Phenolic acids	8277.7	9381	9232.2
Lmhp008718	LysoPC 17:2	Lipids	14434	8366.7	8466.7
mws0057	Eriodictyol 7-O-glucoside	Flavonoids	5221.3	6168.1	5970.6
pmb2799	12,13-EODE	Lipids	8071.2	26761	6156.2
Hmjn008136	Camaldulenic acid	Terpenoids	18176	18752	13955
pmb0962	Lysine butyrate	Amino acids and derivatives	8469.7	10290	15993
mws1078	Anthranilic Acid	Phenolic acids	5985.2	3979.8	7599.8
pmb1096	Indole	Alkaloids	10316	10931	10733

mws2218	Caffeine	Alkaloids	50750	9	9
mws1420	ε-Viniferin	Others	5043.4	3737.3	7308.8
pmd0136	LysoPC 18:0(2n isomer)	Lipids	11440	936.91	5715
pme2237	Dulcitol*	Others	9760.4	5241.1	5509.6
pmf0297	1-Eicosanol	Lipids	9	9029.7	5828.2
Hmbn002174	4-Hydroxyacetophenone	Phenolic acids	9414.6	8499.5	9653.5
Hmcp002268	Limocitrin 3-rhamnoside	Flavonoids	56375	2129.7	2116.8
mws0596	3-Hydroxyanthranilic acid	Organic acids	8077.5	12996	9
pmb2791	9-HpOTrE	Lipids	6307.2	6441.7	6522.9
mws1212	Methyl ferulate	Phenolic acids	3357.3	1925.8	3745.8
pmb2640	Lauric acid	Lipids	6340.5	7446.6	6473.5
mws0289	LysoPE 18:1	Lipids	10266	9	9
pme0500	D-(+)-Melezitose*	Others	3366.2	4115.1	4602.9
pmn001691	9,12,13-Trihyroxy-10,15-octadecadienoic acid	Lipids	9057	5148.3	5159.5
pmn001695	Trihydroxycinnamoylquinic acid	Phenolic acids	16067	9	9
mws0120	Choline alfoscerate	Lipids	5911.3	4759.3	4658
Lmhp008801	LysoPE 18:3	Lipids	19603	9	9
pme2890	L-Homocystine	Amino acids and derivatives	6810.2	4771.2	6668.1
Lmhp009129	LysoPC 15:0	Lipids	6351.6	3189.8	6011.7
mws0636	Phe-Phe	Amino acids and derivatives	3511	4675.1	4582.6
pmb2786	9-HOTrE	Lipids	17809	3054.6	1963.9
pmb1283	L-Glutaminyl-L-valyl-L-valyl-L-cysteine	Amino acids and derivatives	9	9	9

Table S2. Enriched KEGG pathways in endophytic fungi and fungal extracts exposed grape cells.

KEGG Pathways	Metabolite Numbers Enriched						Classification
	C11	C11E	R12	R12E	R32	R32E	
Protein digestion and absorption	2	1	1	1	1	1	Organismal Systems
Longevity regulating pathway					1		
Vitamin digestion and absorption					1		
Metabolic pathways	11	1	3	2	3	2	Metabolism
Biosynthesis of secondary metabolites	4	1	2	1	1	2	
Tyrosine metabolism	1	1	1	1	1	1	
Methane metabolism	1	1	1	1	1	1	
Isoquinoline alkaloid biosynthesis	1	1	1	1	1	1	
Biosynthesis of plant secondary metabolites	2	1	2	1	1	2	
Biosynthesis of alkaloids derived from shikimate	1	1	1	1	1	1	
Flavonoid biosynthesis	1	1	1	1		1	
Microbial metabolism in diverse environments	4		2		1	1	
Caffeine metabolism	1		1		1	1	
Biosynthesis of alkaloids derived from histidine	1		1		1	1	
Phenylpropanoid biosynthesis			1	1	1		
Aminobenzoate degradation	1		1				
Pentose and glucuronate interconversions	1			1			
Ascorbate and aldarate metabolism	1			1			
Amino sugar and nucleotide sugar metabolism	1			1			
Inositol phosphate metabolism	1			1			
Tryptophan metabolism	2						
Phenylalanine, tyrosine and tryptophan	1						
Benzoxazinoid biosynthesis	1						
Lysine degradation	1						
Arginine and proline metabolism	2						
Pyrimidine metabolism	1						
Caprolactam degradation	1						
Purine metabolism	1						
Alanine, aspartate and glutamate metabolism	1						
Arginine biosynthesis	1						
Biosynthesis of antibiotics	1						
2-Oxocarboxylic acid metabolism	1						
Biosynthesis of amino acids	1						
Vitamin B6 metabolism			1				
Cysteine and methionine metabolism			1				
Nicotinate and nicotinamide metabolism					1		
ABC transporters	1						Environmental Information Processing
Neuroactive ligand-receptor interaction	1	1	1	1	1	1	
total counts	31	10	17	15	16	13	
